# Biomarkers in the era of targeted therapy in giant cell arteritis and polymyalgia rheumatica: is it possible to replace acute-phase reactants?

**DOI:** 10.3389/fimmu.2023.1202160

**Published:** 2023-06-15

**Authors:** Guillermo Carvajal Alegria, Mathilde Nicolas, Yannick van Sleen

**Affiliations:** ^1^ EA6295 Nanomédicaments et Nanosondes, Université de Tours, Tours, France; ^2^ Department of Rheumatology, Centre Hospitalier Régional Universitaire (CHRU) de Tours, Tours Cedex, France; ^3^ Department of Rheumatology and Clinical Immunology, University Medical Center Groningen, Groningen, Netherlands

**Keywords:** giant cell arteritis, polymyalgia rheumatica, biomarker, targeted therapy, disease activity monitoring

## Abstract

Research into giant cell arteritis (GCA) and polymyalgia rheumatica (PMR) has become more important in the last few decades. Physicians are facing several challenges in managing the diagnosis, treatment, and relapses of GCA and PMR patients. The search for biomarkers could provide elements to guide a physician’s decision. In this review, we aim to summarize the scientific publications about biomarkers in GCA and PMR in the past decade. The first point raised by this review is the number of clinical situations in which biomarkers could be useful: differential diagnosis of either GCA or PMR, diagnosis of underlying vasculitis in PMR, prediction of relapse or complications, disease activity monitoring, choice, and modification of treatments. The second point raised by this review is the large number of biomarkers studied, from common markers like C-reactive protein, erythrocyte sedimentation rate, or elements of blood count to inflammatory cytokines, growth factors, or immune cell subpopulations. Finally, this review underlines the heterogeneity between the studies and proposes points to consider in studies evaluating biomarkers in general and particularly in the case of GCA and PMR.

## 1 Introduction

Giant cell arteritis (GCA) and polymyalgia rheumatica (PMR) are two inflammatory diseases. Both diseases share a common distinguishing feature: they occur almost exclusively after the age of 50 years. Moreover, GCA and PMR are often associated (1). For this reason, PMR and GCA have sometimes been considered the same disease with different stages. Current understanding of the pathophysiology of the diseases implies shared pathways (2–4).

GCA, as a large-vessel vasculitis, affects the aorta and its branches until the peripheral organs. The disease was first named temporal arteritis in reference to one of its main manifestations: headaches and tenderness of the inflamed temporal artery. In the cranial form of the disease, GCA can also cause jaw claudication, transient or permanent visual loss, diplopia, and tongue ischemia (5–7). But GCA can also have extra-cranial involvement with aortitis, and the symptoms are then less specific (asthenia, fever, weight loss) with sometimes severe complications (limb claudication, mesenteric ischemia, etc.) (8).

PMR is considered an inflammatory rheumatism, affecting the shoulder and pelvic girdle. PMR is responsible for intense pain during the night and prolonged morning stiffness. Even though no severe complications can occur in the absence of associated vasculitis, PMR is responsible for substantial disability in elderly people. Advances in the use of imaging in the disease have underlined the involvement of periarticular inflammation and bursitis (subacromial bursitis, peri-ischiatic bursitis, inter-spinous bursitis) (9–13).

Both diseases are also characterized by a fast—but sometimes only partial—response to glucocorticoids (GC). Nonetheless, the use of prolonged glucocorticoid therapy is now clearly recognized as harmful for patients, and there is an important need for glucocorticoid sparing agents. Translational studies on the pathophysiology of the diseases have led to the discovery of many proteins and cell types involved in the diseases. These discoveries have aided in the selection of potential biomarkers in subsequent studies. Moreover, the development of new technologies, which enabled the screening of a huge number of molecules, led to the identification of many potential biomarkers.

The search for biomarkers has become more important over the years for many diseases. But it is crucial to define the scientific and clinical questions that can be answered by this search. In GCA and PMR, several challenges are still unresolved. First, diagnosis is sometimes difficult for GCA or PMR, and despite the help of new imaging technologies, a simple biological tool could help physicians in their daily diagnostic work-up. Second, it is not always easy to diagnose large-vessel vasculitis in patients with clinical PMR. The involvement of the aorta is often pauci-symptomatic, and simple markers to guide imaging would be useful. Third, disease monitoring is based on clinical symptoms and acute phase reactants. However, better monitoring of biomarkers is required, particularly in light of emerging targeted therapies (e.g., tocilizumab), which can interfere with the measurement of acute phase reactants. Finally, no marker has been clearly associated with a clinical response to GC, methotrexate, leflunomide, or targeted therapies so far.

The aim of this review is to provide the most recent data about the discovery and evaluation of biomarkers in GCA and PMR and to see if acute phase reactants could be replaced by other biomarkers. This review is structured according to clinical situations in which physicians might require the help of biological evaluation to make medical decisions. Without using the methodology of a systematic literature review, we conducted a first search on PubMed for articles that contain the expressions “biomarker” and either “giant cell arteritis” or “polymyalgia rheumatica” in the title or abstract. The search was then extended to references to the identified studies and to articles previously known by the authors.

## 2 Giant cell arteritis

There are still many unanswered questions concerning the management of GCA. Here, we will discuss the potential utility of biomarkers to address three of these questions. i) Because there is no blood test to accurately diagnose GCA, the first question is whether biomarkers can aid in the diagnosis of GCA. ii) Because of the disastrous vascular complications (blindness, stroke), the second question is whether it is possible to use biomarkers to predict complications or relapses of the disease. iii) Finally, the approval for tocilizumab in GCA raised issues about the follow-up of the disease. Therefore, the third question is whether new biological tools can be identified that could monitor disease activity, which would be precious in daily practice.

### 2.1 Biomarkers for diagnosis of giant cell arteritis

The diagnosis of GCA is often challenging. In the cranial form of the disease, with typical manifestations, the diagnosis is typically less complicated. But even in such cases, differential diagnoses have been described (14). The temporal artery biopsy had been considered the gold standard for GCA diagnosis until the most recent recommendations on the use of imaging (15). The use of ultrasound, positron emission tomography, magnetic resonance imaging, and angiography by computed tomography enables a diagnosis of GCA without performing a temporal artery biopsy. This is particularly important for patients with the extra-cranial form of the disease. But diagnosis remains a challenge in first-line care, for physicians who cannot access imaging easily, and in some cases where imaging does not provide a definitive answer and neither does a temporal artery biopsy. To aid the diagnosis, several biomarkers have been investigated (**Table 1**).

**Table 1:** Biomarkers evaluated for the diagnosis of giant cell arteritis.

Category	Biomarker	Usefulness for diagnosis
Daily biology	**CRP** (16–18), **ESR** (17, 18), **leukocytes** (17), **platelets** (17)	Platelet count (17)
Inflammation	**SAA** (16, 18), IL-6 (18), **resistin** (16)	
Vascular involvement	VEGF (16–19), **angiopoietin 1** (17, 18), **angiopoietin 2** (17, 18), sTie2 (17, 18), VCAM-1 (16)	
Monocytes/macrophages involvement	**YKL-40** (16, 17, 20), **sCD206** (17), GM-CSF (21), **MCP-3** (22), M-CSF (16), MARCO (16)	MCP-3 (for prediction of GCA development)
Extracellular remodeling	MMP-1 (16), MMP-2 (16), MMP-3 (17), MMP-9 (16, 17), **A1AT** (17)	MMP-3 (17)
Neutrophil involvement	**Calprotectin** (17), **PR3** (17), **elastase** (17)	PR3 (17)
Lymphocyte activation and cytokines	**BAFF** (16, 21), INFα (21), IFNγ (16, 21), IL-1β (16, 21), IL-2 (16, 21), IL-4 (21), IL-5 (21), **IL-6** (16, 21), IL-7 (21), IL-8 (16, 21),IL-9 (16), **IL-10** (16, 21), IL-12 (21), IL-13 (16, 21), IL-15 (21), IL-17 (16, 21), IL-18 (16), IL-23 (16), IL-27 (16), IL-31 (16), TNFα (16, 21), TNF-R1 (16), sIL-1Ra (21), **sIL-2R** (21)	
Cell migration	**CCL2** (21), CCL3 (21), CCL4 (21), CCL5 (21), **CCL11** (21), **CXCL9** (21), CXCL10 (21)	

Biomarkers described as significantly different compared to healthy controls are in bold.

#### 2.1.1 Acute-phase reactants

Acute-phase reactants, such as C-reactive protein (CRP), or erythrocyte sedimentation rate (ESR), are routinely used as tools for diagnosis and monitoring disease activity. But the diagnostic value of CRP or ESR has been discussed in several studies (17, 23–26). Sensitivity of CRP and ESR was reported at 96.2% and 91.5%, respectively for the diagnosis of temporal artery positive GCA compared to a negative temporal artery biopsy (suspected GCA) (26). But their specificity was poor (respectively, 41.3% and 37.4%). In a large study on biomarkers, the area under the curve for the diagnostic performance of CRP was 0.63, which does not indicate good discrimination (17). In another large cohort, the sensitivity of CRP and ESR to predict a positive temporal artery biopsy was found to be 86.4% and 84.2%, respectively (23). But the specificity was much lower (30.5% for CRP and 29.5% for ESR). So, as expected, the negative predictive value is quite acceptable for CRP and ESR, but the positive predictive value is low. Serum amyloid A, though not performed routinely in every center, has also been studied and was found to be elevated in GCA patients compared to blood donors in two different studies (16, 27). But no threshold and no diagnostic performance were defined for its use in daily practice.

ESR and CRP should be used to rule out the diagnosis of GCA (except for patients with very typical symptoms requiring more investigation), but they are not sufficient to validate the diagnosis of GCA.

#### 2.1.2 Full blood count

Platelets and other parameters of the full blood count are highly affected by inflammation. Several studies evaluated the use of platelet counts as a diagnostic tool for GCA (17, 24–26). In receiver operating characteristic (ROC) analysis, the area under the curve could range from 0.66 to 0.75. Two studies compared patients with a positive temporal artery biopsy to patients with suspected GCA but a negative temporal artery biopsy (24, 26). Another study compared GCA patients to mimics of GCA (17). A high platelet number indicated a higher probability of GCA compared to the mimics (17). Platelet count could not be used alone because its specificity was not high enough. But a low platelet count could be a trigger to look further into other diagnoses.

One study focused on the neutrophil to lymphocyte ratio, platelet to lymphocyte ratio, and monocyte to lymphocyte ratio to diagnose GCA (24). The sensitivity and specificity of these ratios were not reported, and the areas under the curves of ROC analyses were between 0.55 and 0.62.

The platelet count is currently the best biomarker available that is complementary to CRP and ESR. The threshold should be considered between 350 and 400.10^9^/l.

#### 2.1.3 Autoantibodies

Several autoantibodies, such as anti-cardiolipin autoantibodies and anti-ferritin autoantibodies, have been studied in GCA. Nevertheless, in the end, the diagnostic performance was not good enough to allow the use of such autoantibodies in daily practice (28–32). Considering the specificity of autoantibodies as diagnostic tools or to assess disease activity, we will not go into detail in this review. Data about autoantibodies in GCA have been reviewed previously elsewhere (33).

#### 2.1.4 Monocyte chemoattractant protein 3

Very interesting data are provided by a Swedish study on the biomarkers that were measured in people prior to the development of GCA (22). In this study, the authors used a large cohort of 30,447 patients—initially developed to study cancer—and identified 94 cases of incident GCA. The authors analyzed 92 inflammatory biomarkers and observed that interferon gamma and monocyte chemoattractant protein 3 (MCP-3) were associated with a greater risk (an odds ratio of 3.74 in the 8.5 years before the onset of the disease) to develop GCA, several years before the appearance of the first symptoms. Although no analysis was made to estimate the potential diagnostic usefulness of those markers and only MCP-3 remained significant in a sensitivity analysis, they might be promising biomarkers. The involvement of monocytes and macrophages has been more and more studied in GCA (34). Their role in the pathophysiology of the disease might precede the appearance of clinical symptoms. So, early identification of monocyte disturbances might help diagnose GCA earlier.

The usefulness of MCP-3 should be evaluated in other studies.

#### 2.1.5 Cytokines and other proteins

In a study comparing 97 untreated GCA patients to 53 healthy controls (16), many markers were differentially expressed between the two groups. The identified proteins are involved in several pathways already identified in the pathophysiology of GCA, such as inflammation (interleukin [IL]1 beta, IL-6, IL-8, IL-23, IL-31, interferon gamma, etc.), vascular involvement (vascular endothelial growth factor, intercellular adhesion molecule 1), or monocyte/macrophage involvement (macrophage receptor with collagenous structure, macrophage colony stimulating factor, chitinase 3-like protein 1, also known as YKL-40). In addition to the interesting results of their clustering analysis, three candidates for biomarkers are identified by the authors: serum amyloid A, IL-6, and IL-23. As we will detail later in this review, IL-6 and IL-23 have also been studied as biomarkers for relapse. Finally, YKL-40 serum levels were also found to be increased in patients with GCA compared to controls. The role of YKL-40 in the pathophysiology of GCA is not clearly understood yet. Nevertheless, recent data suggest that YKL-40 is strongly expressed by GM-CSF-skewed, pro-inflammatory macrophages in the temporal arteries of GCA patients (35). YKL-40 likely plays a role in mediating the formation of small new vessels and in local tissue destruction through matrix metalloproteinase (MMP)-9 production. In another study analyzing a large number of candidates for biomarkers, YKL-40 was found to be elevated compared to healthy controls but not compared to patients with a pathology mimicking GCA (17). In another study comparing GCA patients to healthy controls, B cell activating factor (BAFF), chemokine C-X-C motif (CXCL)9, and IL-6 showed great performances (with area under the curve of respectively 0.90, 0.93, 0.85, and 0.98 in ROC analysis).

Osteopontin has also been evaluated in order to differentiate patients with vasculitis but a negative temporal artery biopsy from patients without vasculitis and a negative temporal artery biopsy in a cohort of patients with negative temporal artery biopsy with a suspicion of GCA (36). Osteopotin was strongly expressed in all patients with a positive temporal artery biopsy and was not detected in patients with a negative temporal artery biopsy without vasculitis. But in the group of patients with a negative temporal artery biopsy and vasculitis, osteopontin was detected in only two out of 17 patients. These data do not suggest that osteopontin is useful for the diagnosis of GCA in addition to the pathological examination of temporal artery biopsies.

The choice of a comparator in studies evaluating the utility of biomarkers seems critical. Indeed, when GCA patients are compared to healthy controls without any inflammatory disease, we probably collect data useful to understand the pathophysiology of the disease. But, in clinical practice, biomarkers for diagnosis will be used in patients with clinical manifestations (vasculitis symptoms, fever, elevated acute-phase reactants). The performance and utility of biomarkers must be evaluated in the context of clinical reasoning. Consequently, to evaluate a biomarker useful for diagnosis in difficult cases or in patients with symptoms suggestive of GCA, biomarkers should be measured in cohorts including patients with GCA and patients with mimicking diseases.

IL-6 has been reported as a potential diagnostic biomarker in several studies and has a strong correlation with CRP and ESR. Nevertheless, IL-6 cannot be measured in daily practice in most countries.

### 2.2 Biomarkers for relapsing diseases and complications

#### 2.2.1 Biomarkers to follow disease activity

##### 2.2.1.1 Acute-phase reactants and hematology parameters

ESR, CRP, hemoglobin, and platelets have been evaluated as markers of relapse in GCA patients treated with GC. Most relapses occurred in patients with less than 5 mg of GC. CRP and ESR were studied in relapsing and non-relapsing patients with GCA during the first 3 months, the remaining portion of the first year of treatment, and the period after the first year of treatment (37). ESR was significantly higher in relapsing patients during the three periods studied. CRP was not significantly higher between relapsing and non-relapsing patients during the first three months of treatment.

So, ESR might be a better marker of relapses during the first 3 months of treatment. The daily GC dose might play a role in CRP’s ability to flag disease activity.

##### 2.2.1.2 Interleukin 6

The first reports on the correlation between IL-6 serum or plasma levels and disease activity or inflammation in GCA patients are from the early 90s (38, 39). In the first study, Dasgupta and Panayi described an increase in IL-6 in the serum of 12 PMR patients and 3 GCA patients. IL-6 levels were increased in untreated patients compared to healthy controls. In contrast, the levels of IL-6 in the serum of treated patients were not increased. Moreover, IL-6 levels were correlated with ESR (38). In the second study, Roche et al. identified higher IL-6 levels in the plasma of 13 untreated PMR and 19 untreated GCA patients compared to 20 healthy controls. The authors also identified a correlation between IL-6 plasma levels and ESR (39). IL-6 serum levels might also be strongly influenced using GC. In a study on biomarkers (including IL-6) evaluating two kinds of GC, IL-6 was measured after four weeks of GC (40). In this study, the observed IL-6 levels were not as high as expected in patients with active GCA. Therefore, knowledge of the kinetics of the cytokines under specific therapies is essential before implementing the use of these cytokines in the monitoring of disease. In other studies, serum levels of IL-6, but not soluble IL-6 receptors, were associated with disease activity (41). The IL-6 receptor levels were slightly increased in PMR patients at onset compared to healthy controls. Moreover, IL-6R levels were stable in PMR patients during follow-up. Interestingly, IL-6 levels were found to be associated with inflammation on positron emission tomography. So, IL-6 levels might reflect the intensity of vascular uptake in positron emission tomography.

##### 2.2.1.3 Interleukin 12 and 23

Among the cytokines studied in the pathophysiology of GCA, IL-12 and 23 have been reported as relevant in the development of vascular inflammation (42). Both cytokines were able to increase the production of IL-6 in explants of the temporal arteries in GCA patients. The assumed role of IL-23 in the pathophysiology of the disease has recently led to the evaluation of the efficacy of IL-23 inhibition in GCA (ustekinumab NCT03711448 and gulsekumab NCT04633447). IL-23 was also evaluated as a potential biomarker in a prospective and longitudinal study on 31 untreated patients with active GCA who started GCA treatment with or without leflunomide (20). Serum IL-23 levels were high in patients at the time of a disease relapse, and the IL-23 levels had been increasing since the visit prior to the relapse, when the disease was not active. Even though IL-23 has not been properly evaluated as a predictor of a relapse in GCA, these data suggest that it might be of interest. The use of IL-23 inhibitors in clinical trials will probably give us more data about the role of this cytokine in GCA and its potential utility as a biomarker.

##### 2.2.1.4 The interferon pathway

Interferon pathways have also been studied in GCA and could potentially serve as biomarkers of disease activity. Inflamed aortas of GCA patients displayed a more pronounced interferon signature than non-inflammatory aorta tissue (43). Also, the level of interferon-alpha in the serum of patients with an active disease was higher than in patients with an unactive disease. The performance of the level of interferon used to monitor disease activity has not been evaluated *per se*. But it seems that only three out of fifteen patients with a disease in remission had interferon alpha levels over 0.02 pg/ml, whereas this was the case in more than half of patients with an active disease.

##### 2.2.1.5 The Janus kinase/signal transducer and activator of transcription pathway

In the same study (43), the authors reported an upregulation of the cytokines and chemokines pathways, but also of the Janus kinase/signal transducer and activator of transcription (JAK/STAT) pathway. The involvement of the JAK/STAT pathway is of interest because several inhibitors of this pathway have been developed in the last decade. It has been demonstrated that tofacitinib, an inhibitor of JAK1 and JAK3, reduced inflammation due to T-cell activation in inflamed arteries grafted to immunodeficient mice (44). Recently, baricitinib, another JAK inhibitor, has been evaluated in relapsing GCA in an open-label study (45). Baricitinib was well tolerated, and most of the patients on baricitinib were able to stop GC. Finally, one large clinically controlled trial is ongoing, evaluating the efficacy of upadacitinib in CGA patients (NCT03725202). Whether biomarkers reflecting JAK/STAT pathway activation can be used to monitor disease activity is still unclear.

Though IL-6, IL-12, IL-23, and interferons might seem promising, the main limitation to their use in daily practice is, once again, the possibility of performing a routine analysis.

#### 2.2.2 Biomarkers to evaluate prognosis measured at diagnosis

Novel biomarkers that could predict whether GCA patients are prone to disease complications or whether they are easy or difficult to treat are highly anticipated and could potentially be used for treatment stratification. Early studies mainly focused on clinical criteria to predict complications in GCA (46). Hyperlipidemia at diagnosis, for example, was found to be associated with a higher risk of aortic aneurysms or aortic dissection, whereas an increased ESR at diagnosis was associated with a lower risk of large vessel stenosis (46, 47). Another study, however, showed that patients with ischemic complications were reported to have lower levels of IL-6 in both the serum and in the temporal arteries at diagnosis compared to patients without complications (48).

There are two studies that aim to predict the response to GC at diagnosis using a score of systemic inflammation containing both common inflammatory parameters and disease symptoms. Hernández-Rodríguez et al. suggested that patients with three criteria among fever, weight loss, ESR ≥85 mm/h, or hemoglobin <11 g/dl needed a longer use of GC with a higher cumulative dose and more relapses (49). Similarly, Nesher et al. made use of a score containing ESR, hemoglobin, leukocyte, and thrombocyte levels in addition to the presence of fever to predict a favorable or unfavorable response to GC treatment (50). Validation studies are required to identify which scores perform better in independent cohorts.

It has been suggested that the expression of IL-17A in the vasculitis lesions of the temporal arteries in GCA patients might predict a good response to GC (51). The expression of IL-17A mRNA in the temporal arteries of patients who achieved sustained remission or had only one relapse was higher than in patients with more than one relapse. Moreover, patients with high levels of IL-17A mRNA expression were able to stop prednisone earlier than others. The role of IL-17A in GCA and PMR is currently under the spotlight due to the recent demonstration of the efficacy of secukinumab, an anti-IL-17A monoclonal antibody (52).

In a study analyzing data from epigenome and transcriptome-wide associations in CD14+ monocytes from patients affected by GCA, many pathways were differentially regulated in GCA patients compared to controls, but also in GCA patients treated with GC compared to GCA patients without GC (53). The authors suggest that CD163, a receptor expressed by monocytes with anti-inflammatory properties, could represent a potential biomarker for the response to GC in GCA.

YKL-40 has also been evaluated as a potential marker of vascular complications (20). In this cohort, YKL-40 serum levels were associated with vessel occlusion and trans-arterial inflammation. Inflammation and occlusion were assessed in the pathological evaluation of temporal artery biopsies. Even though not all patients with an inflamed temporal artery biopsy had elevated YKL-40 serum levels (lack of sensitivity), a level of YKL-40 over 100 ng/ml seems highly specific for inflammation. The level of inflammation might be associated with a higher risk of vascular complications. But no proper demonstration of the association between YKL-40 and vascular complications has been provided so far. Additionally, high YKL-40 levels at diagnosis were found to be predictive of a longer time of GC-free remission in another study (18).

At the inflammatory site, new outgrowth of small vessels can be observed in GCA patients, and in the blood, angiogenesis markers reflecting these processes could potentially serve as prognostic markers. High serum levels of vascular endothelial growth factor and angiopoietin-1 were found to be protective, as these patients had a shorter time to GC-free remission than patients with low serum levels. High levels of angiopoietin-2, however, tended to be associated with a longer GC treatment duration (18).

Osteopontin levels have also been described as being associated with the risk of relapse (54). Osteopontin is involved in bone and cartilage physiology but has also been described as playing a role in the immune response. In this study, patients with more than one relapse had higher levels of osteopontin at baseline. Moreover, patients with the highest level of osteopontin required longer glucocorticoid therapy.

So far, the most commonly studied predictors of the cumulative dose of GC are still the inflammatory parameters, either CRP alone (55) or the association of several parameters (fever, weight loss, ESR, hemoglobin, leukocytes, and thrombocytes).

**Table 2** summarizes the biomarkers to predict flares during GCA that we evaluated here. Some markers—mainly the common ones—have been studied in several cohorts, and the results might be controversial. CRP and ESR are the archetypes of this problem. Those markers have been evaluated in many studies and are sometimes associated with a higher risk of relapse, sometimes not. Some biological markers have also been evaluated in temporal artery biopsies.

**Table 2:** Biomarkers evaluated to predict relapse in giant cell arteritis.

	Decreased risk of relapse	No impact on the risk of relapse	Increased risk of relapse
Serum/plasma	Hemoglobin (56, 57) MMP-2 (16)	CRP (29, 56, 58–61) ESR (54, 56–60, 62, 63) Fibrinogen (57) Hemoglobin (54, 58, 60, 63, 64) Leukocytes (62, 63) Ferritin (64) IL-6 (54, 64) Platelets (56, 60, 62–64)	CRP (16, 54, 57, 64) ESR (16, 37, 64, 65) Fibrinogen (64) Leukocytes (64) Haptoglobin (58, 64) Osteopontin (54) SAA (16, 64) Anticardiolipin antibodies (29)
Temporal artery biopsies		Giant cells (66, 67) IL-1β (68) IL-6 (68)	Giant cells (56) IL-17 (51) TNF (68) High inflammation (56) Intraluminal thrombosis (56)

#### 2.2.3 Biomarkers to predict a relapse or a complication measured during follow-up

In 2010, the serum levels of IL-6 and tumor necrosis factor (TNF) alpha in GCA patients were assessed for a potential role in predicting relapses or complications in GCA patients in remission (69). The levels of both cytokines were increased compared to controls, but they were even higher in patients with a previous relapse of the disease than in patients without. However, these cytokines were not associated with the development of vascular complications of the disease.

Among the family of acute-phase reactants, pentraxin 3 has also been found to be increased in GCA patients with recent optic nerve ischemia (19, 70). Although pentraxin 3 is considered an acute-phase reactant, it was not found elevated in patients with PMR (71). Indeed, pentraxin 3 has been linked to vascular inflammation (72), but no studies have evaluated its role in predicting underlying vasculitis in PMR patients.

Autoantibodies directed against the receptor for endothelin-1 may also predict future vascular complications in GCA patients. Endothelin-1 is a peptide able to induce vascular contraction. Endothelin-1 expression is upregulated in temporal artery biopsies from patients with active GCA (73–75). The use of macitentan, an antagonist of endothelin-1, reduced the proliferation of vascular smooth muscle cells in patients with GCA (76). Whereas endothelin-1 was mostly measured in temporal arteries, levels of autoantibodies directed against endothelin-1 receptor A were studied in the serum of GCA patients (77). The authors showed that autoantibody levels under 2.023 U/ml were associated with an increased risk of vascular complications in the following 8 weeks.

Another study aimed to identify biomarkers for aortic dilatation in patients with inactively treated GCA (78). The authors measured the white blood counts, acute-phase reactants, cytokines (IL-5, IL-8, IL-10, IL-17A, IL-18, IL-1RA, and TNFα), interferons (alpha and gamma), selectins (L-selectin and P-selectin), platelet-derived growth factor, interferon gamma-induced protein 10, and soluble intercellular adhesion molecule 1. None of the investigated markers were differentially expressed between GCA patients with and without aortic dilatation, with 20 patients per group. Some markers were not detected and therefore not analyzable (IL-5, IL-10, IL-17A, IL-1RA, TNFα, interferons α, and γ). No significant difference was observed for the other markers.

**Figure 1:**
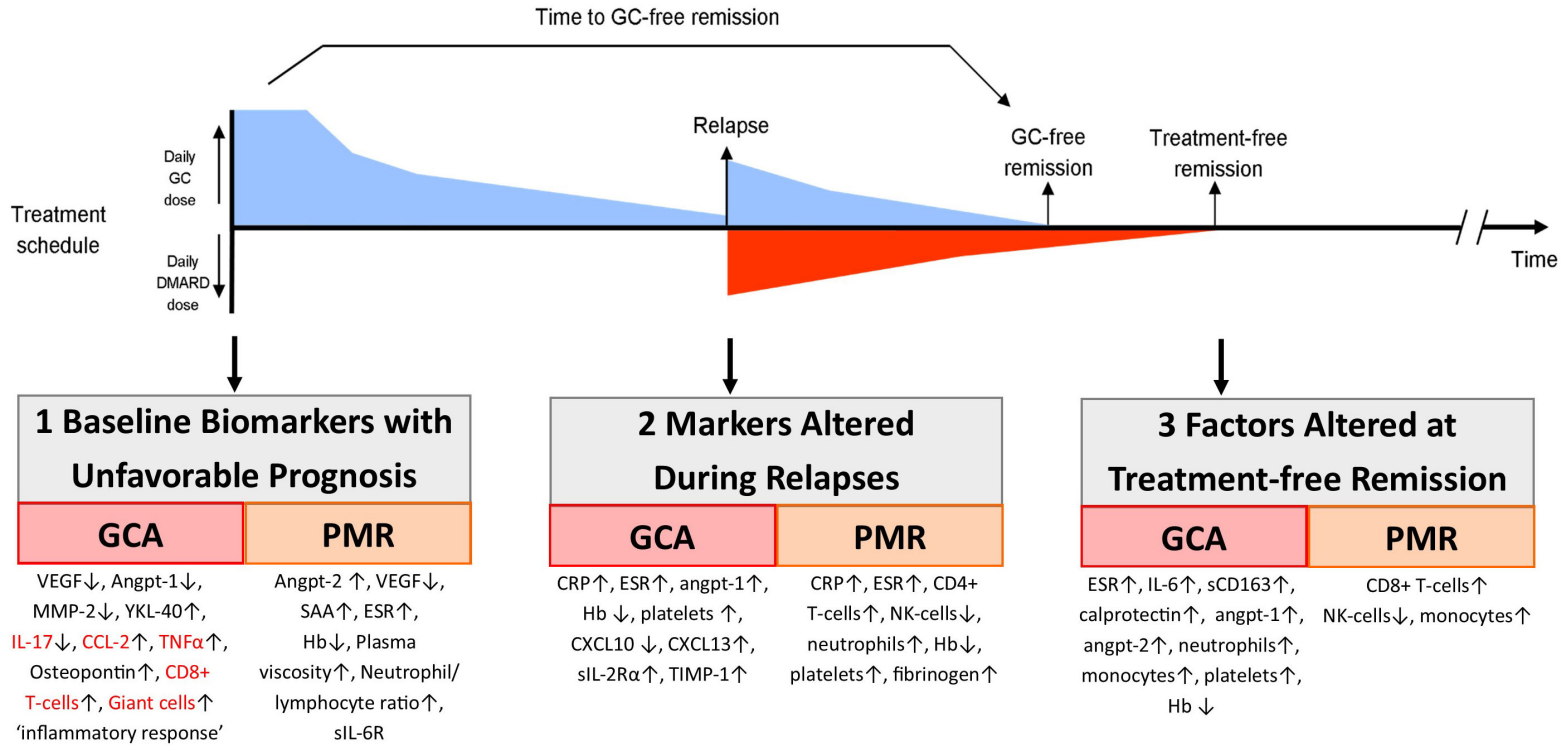
Biomarkers important during follow-up of GCA and PMR patients. Here we show the timeline of the treatment of an example patient that receives glucocorticoid (GC) treatment, which is tapered until the patient relapses. At that moment, the GC dose is increased and a DMARD is added. At some point later, the patient reaches GC-free and also treatment-free remission. In this figure, we also show at which point biomarkers could aid the monitoring of patients (1): are biomarkers measured at diagnosis that are predictive of an unfavorable (i.e., relapsing) disease course (2); are biomarkers that are altered during clinical relapses; and (3) are biomarkers that remain altered in patients in treatment-free remission, potentially reflecting smoldering tissue inflammation. Biomarkers in red are measured in temporal artery biopsies; others are measured in blood.

**Figure 1** shows the potential biomarkers at three critical points in the evolution of GCA: prognosis at diagnosis, evaluation of relapses, and evaluation of underlying activity in apparent treatment-free remission.

### 2.3 Biomarkers for the monitoring of targeted therapies

Considering the burden of long-term GC in the treatment of GCA (79, 80), other options have been investigated as first-line or as GC sparing agents. Tocilizumab is a monoclonal antibody targeting the membranous and soluble receptors of IL-6. Increased levels of IL-6 have been described in untreated and relapsing GCA (69), leading to the evaluation of tocilizumab in GCA. Tocilizumab was first evaluated in case series (81–84) before being evaluated in a large randomized controlled trial known as the GIACTA trial in 2017 (85). Tocilizumab is now the first targeted therapy approved by GCA. Given that IL-6 is an essential stimulator of acute-phase markers such as CRP, monitoring disease activity is not possible using these markers in patients using tocilizumab (86). ESR was also reported at very low levels under tocilizumab therapy and did not increase in most patients during flares (86). Results from the Spanish cohort of GC patients treated with tocilizumab also showed a dramatic decrease in both CRP and ESR (87, 88). Therefore, novel IL-6-independent biomarkers that reflect vessel wall inflammation are needed for monitoring these patients.

In an ancillary study of the GIACTA trial, the team from Boston described disturbances in the T cell compartment of patients included in the study (89). In this study, they described an inflammatory profile of regulatory T cells secreting IL-17 and associated with a less functional isoform of the Foxp3 transcription factor. Tocilizumab enabled a better correction of the regulatory T cell abnormalities. But those analyses have not been viable in daily practice so far, and these data remain exploratory for therapeutic monitoring. In a small study including 26 patients, a decline in IL-6 serum levels during treatment was associated with a decreased risk of relapse after the cessation of tocilizumab therapy (90).

Osteopontin has been suggested as a potential biomarker to monitor disease activity under tocilizumab therapy. Indeed, in the previously cited study evaluating the utility of osteopontin in the prediction of flares, the level of osteopontin in GCA patients treated with GC or with tocilizumab was also evaluated (54). As expected, the level of CRP was very low or undetectable in the serum of patients treated with tocilizumab. However, osteopontin was still detected in the serum of patients treated with tocilizumab. More data are mandatory, especially to assess whether osteopontin levels reflect flares in patients on tocilizumab therapy, in order to consider its use in clinical practice.

Recently, the utility of complements (C3 and C4) in the monitoring of disease activity was assessed in a retrospective cohort (91). Although neither C3 nor C4 were elevated at the time of diagnosis compared to unaffected controls, their levels decreased after the initiation of GC treatment. Moreover, in this cohort, nine patients were treated with tocilizumab. The decrease in both C3 and C4 was more pronounced in patients treated with tocilizumab than in the patients treated with GC. The levels of C3 and C4 were not correlated to the levels of the CRP or ESR, suggesting that complement might be independent from acute-phase reactants and might be useful to assess disease activity under tocilizumab therapy. When tocilizumab was stopped, the levels of both C3 and C4 increased. Future studies should indicate whether C3 and C4 can be used as monitoring biomarkers in GCA patients on tocilizumab treatment. Only a little data is available about the utility of complements in GCA. The first report is from 1986 and gives mainly information about pathophysiology (92). A more recent study evaluated the utility of complement in Takayasu arteritis (93). Because of clinical similarities, Takayasu arteritis and GCA might share common pathophysiological pathways. But it remains unclear if the involvement of complement is specifically associated with large vessel injuries or is a consequence of inflammatory processes.

Osteopontin, C3, and C4 could be biomarkers useful to assess disease activity under tocilizumab therapy, but more studies are needed to confirm this.

### 2.4 Biomarkers reflecting underlying inflammation

There is increasing evidence that GCA (and PMR) symptoms can readily return due to persistent inflammation at the tissue level. Measures of systemic inflammation are suppressed in treated patients but do not necessarily reflect ongoing tissue inflammation. Observations by ultrasound imaging show that vessel-wall thickening persists for years in GC-treated GCA patients (94). Maleszewski et al. performed a follow-up temporal artery biopsy in GCA patients that had biopsy-proven GCA (95). Most patients showed persistent vascular inflammation with macrophages and T cells after up to one year of GC treatment. This number of patients with persistent inflammation is likely even an underestimation, as the follow-up biopsy was taken on the opposite site of the primary biopsy. Inflamed tissues may be missed due to skip lesions, and vascular inflammation may be present in the aorta and its branches (which may be even more GC-resistant (96)). Interestingly, it has been shown that two macrophage-produced proteins remain elevated in the serum of GCA patients during the first year of treatment (35). Calprotectin and YKL-40, released by infiltrating phagocytes and CD206+ macrophages, respectively, may thus possibly reflect tissue inflammation. Calprotectin and YKL-40 are still expressed in aorta biopsies of patients with GCA-caused aneurysms, a complication typically representing late-stage disease. This is in accordance with leukocyte subset data, showing that the myeloid bias is not corrected by GCs and is still apparent in treatment-free GCA and PMR patients (37).

## 3 Polymyalgia rheumatica

PMR remains an understudied disease. Compared to other rheumatic diseases or GCA, the number of available publications on biomarkers was scarce. Nevertheless, during the last decades, research on PMR has evolved and led to new diagnostic, imaging, and therapeutic aspects of the disease. Several challenges have been highlighted, such as the identification of asymptomatic vasculitis in PMR patients, optimization of GC therapy, and identification of alternatives to GC (mainly with targeted therapies but also with methotrexate and leflunomide). For now, the impact of subclinical vasculitis on treatment is not determined, and no treatment has been approved for PMR despite several randomized controlled trials demonstrating the efficacy of IL-6 blockade (97, 98). But these questions are what is at stake for tomorrow and lead us to the same consideration of the utility of biomarkers as in GCA.

### 3.1 Biomarkers for the diagnosis of PMR

The identification of biomarkers for the diagnosis of PMR has not been studied extensively. One study compared PMR patients to healthy controls and provided sensitivity and specificity of 100% for IL-6 levels over 8 pg/ml in the serum (21). BAFF and CXCL9 and 10 also had excellent performances. But a comparison with patients with suspected PMR (and possibly another inflammatory condition) is lacking.

The current biomarkers identified represent clues to the pathophysiology of the disease rather than clinical tools.

### 3.2 Biomarkers for the identification of underlying vasculitis in polymyalgia rheumatica

An important issue at the time of PMR diagnosis is whether the patient has an overlapping GCA. This is important, as complications of GCA can be dangerous, such as blindness and aneurysms. GCA patients also require a higher starting glucocorticoid dose. Arterial inflammation in patients with PMR manifests itself mostly in the aorta and its branches (99), and this type of GCA (LV-GCA) has mostly no specific symptoms. The links between PMR and GCA are not clear yet. Despite a strong association between both diseases, we do not understand how and why so many PMR patients will develop concomitant vasculitis (1). The use of ultrasonography and positron emission tomography has led to the identification of vascular involvement in patients with PMR but without any clinical signs of vasculitis (1, 100, 101). However, the use of ultrasonography or positron emission tomography is either time-consuming or expensive and is not available to all physicians. The use of simple biomarkers might be more suitable to enable a large screening of all patients with PMR. However, both GCA and PMR patients have high levels of acute-phase markers, rendering them mostly useless in making a distinction between these two diseases.

There are a few potential biomarkers that could aid in the identification of those PMR patients who also have overlapping GCA at diagnosis. In 2015, a first study described an increase in MMP-3 levels in the serum of patients with isolated PMR compared to patients with PMR and concomitant GCA (102). In this study, the threshold for MMP3 was determined at 140 ng/ml. Sensitivity and specificity were then 91% and 66%, respectively. A recent study validated data from earlier studies that point to the utility of vascular remodeling markers to identify patients with PMR + GCA (17). Low levels of MMP-3 and a high angiopoietin-2/1 ratio were found to be accurate biomarkers for GCA in patients with PMR in both a Dutch and a Danish cohort. High ESR levels could also discriminate between PMR patients with and without GCA in both cohorts, albeit with lower accuracy. Additionally, low levels of calprotectin, which is considered a marker of neutrophil and monocyte migration, were found to be associated with overlapping GCA in PMR patients (103). **Table 3** summarizes studies evaluating biomarkers for the detection of underlying vasculitis. As we discussed above, pentraxin 3 could be a good discriminatory marker, as it was found elevated in GCA but not in PMR patients, and because it might be associated with vascular inflammation. But further studies are needed to evaluate its usefulness.

**Table 3:** Biomarkers evaluated to detect subclinical vascular involvement in polymyalgia rheumatica.

Molecule	References	Population	Threshold	Interest
CRP	van Sleen et al. (17)	Suspicion of GCA or PMR		
ESR	van Sleen et al. (17)	Suspicion of GCA or PMR	60–91 mm/h	GCA/PMR Vs Isolated PMR
Leukocytes	van Sleen et al. (17)	Suspicion of GCA or PMR		
Platelets	van Sleen et al. (17)	Suspicion of GCA or PMR		
MCP-1	Ellignsen et al. (104)	Untreated PMR/GCA		No difference between PMR and GCA
VEGF	van Sleen et al. (17)	Suspicion of GCA or PMR		
Angiopoietin 1	van Sleen et al. (17)	Suspicion of GCA or PMR	Agpt1/2 ratio 0.048–0.051	GCA/PMR Vs Isolated PMR
Angiopoietin 2	van Sleen et al. (17)	Suspicion of GCA or PMR	Agpt1/2 ratio 0.048–0.051	GCA/PMR Vs Isolated PMR
Soluble Tie2	van Sleen et al. (17)	Suspicion of GCA or PMR		
YKL-40	van Sleen et al. (17)	Suspicion of GCA or PMR		
MMP3	van Sleen et al. (17)	Suspicion of GCA or PMR	14–23 ng/ml	GCA/PMR Vs Isolated PMR
MMP9	van Sleen et al. (17)	Suspicion of GCA or PMR		
sCD206	van Sleen et al. (17)	Suspicion of GCA or PMR		
Calprotectin	van Sleen et al. (17)	Suspicion of GCA or PMR		
PR3	van Sleen et al. (17)	Suspicion of GCA or PMR		
Elastase	van Sleen et al. (17)	Suspicion of GCA or PMR		
A1AT	van Sleen et al. (17)	Suspicion of GCA or PMR		

ESR, MMP-3, and the angiopoietin 1/angiopoietin 2 ratio demonstrated their abilities to identify the underlying GCA in PMR patients. But only ESR is currently available in daily practice in most countries.

### 3.3 Biomarkers for the risk of relapse of PMR and disease activity

One study analyzed sIL-6R and sgp130 as biomarkers to predict the risk of relapse in PMR (105). The authors found a positive correlation between the sIL-6R concentration in the serum of patients at diagnosis and the number of relapses. The same correlation was observed between the serum level of sIL-6R after 1, 3, and 12 months of treatment and the number of relapses. Since 2008, no study has replicated these results.

A correlation between several parameters from the full blood count and ESR or CRP has been reported. The platelet-to-lymphocyte ratio seems to be correlated with both CRP and ESR (106). The neutrophil-to-lymphocyte ratio could also be a predictor of poor outcomes, but no threshold has been determined yet (106, 107). IL-6 has also been reported to have a good correlation with CRP, ESR, leukocytes, platelets, and neutrophils (21, 108). Surprisingly, TNFα also correlated with ESR and haptoglobin but not to CRP (49). Finally, vascular endothelial growth factor and BAFF were also reported with a good correlation to both ESR and CRP (21, 109).

The strong correlation observed between CRP (or ESR) and several biomarkers is promising, but none of these biomarkers can be used currently.

### 3.4 Biomarkers for the prognosis of polymyalgia rheumatica under conventional or targeted therapies

#### 3.4.1 Glucocorticoids and synthetic glucocorticoid sparing agents

Similarly to GCA, biomarkers that can predict disease course are highly anticipated for PMR patients. Several markers have been postulated that could aid in the stratification of PMR patients. A high ESR at diagnosis has been associated with long-term glucocorticoid use during follow-up in two studies (110, 111), although another study could not find this association (37). Angiopoietin-2, which was also associated with concomitant GCA in PMR patients, was also a strong predictor of glucocorticoid treatment duration in PMR patients with isolated disease (18). Other potential markers of an unfavorable disease course are a high neutrophil-to-lymphocyte ratio, a high red blood cell distribution width, and a low hemoglobin (37, 107, 112). CRP is routinely used to assess inflammatory activity. The decrease of CRP in the blood after one month of GC therapy might be predictive of remission with GC and of a lower cumulative GC dosage (55).

Full blood count elements and serial CRP measurements are still the main predictors of the response to GC.

#### 3.4.2 Targeted therapies

As discussed above in GCA, CRP is hardly interpretable in patients using tocilizumab treatment (113). In PMR, too, biomarkers would be needed during the use of IL-6 blocking therapies. The level of gammaglobulins before tocilizumab initiation has been suggested to be predictive of early response to tocilizumab therapy (108). But these results have not been reproduced yet. **Table 4** summarizes the exploratory markers evaluated in PMR.

**Table 4:** Exploratory biomarker in polymyalgia rheumatica.

Molecule	References	Population	Threshold	Interest
Hemoglobin	Pulsatelli et al. (105)	PMR onset and under GC therapy	11.5 g/dl	Increase of the risk of relapse under GC
MCP-1	Ellignsen et al. (104)	Untreated PMR/GCA		No difference between PMR and GCA
VEGF	Meliconi et al. (109)	Untreated and treated PMR		
MMP3	Ribbens et al. (114)	Inflammatory diseases		MMP3 is higher in diseases with synovial involvement including PMR
CXCL9	van der Geest et al. (21)	Early untreated PMR or GCA		Increased in PMR Correlated moderately with ESR and CRP
IL-6	van der Geest et al. (21)	Early untreated PMR or GCA		Increased in PMR Decreased in GC induced remission Correlates with CRP and ESR
IL-6	Carvajal Alegria et al. (115)	Early untreated PMR then under tocilizumab therapy		Increased in PMR Correlates with CRP IL-6 decrease associated with B-cell increase
Gammaglobulins	Carvajal Alegria et al. (108)	Early untreated PMR then under tocilizumab therapy		Associate with earlier response to tocilizumab
Serum IL-6R	Pulsatelli et al. (105)	PMR onset and under GC therapy	56 ng/ml	Increase of the risk of relapse under GC

## 4 Discussion

Several studies evaluated biomarkers in GCA and PMR. Due to differences in the methodology, many molecules studied could not be used in daily practice. Moreover, the availability of a validated assay for daily care limits the use of some promising biomarkers. **Tables 5**, **6** summarize the data from ROC analysis and correlation studies in GCA and PMR. To summarize this review,

- For the diagnosis of GCA, ESR and CRP are useful to rule out the diagnosis in the case of values in the normal range. The platelet count can also be used in the diagnostic process, with a threshold defined, depending on the study, between 350 and 400.10^9^/l. MCP-3, IL-6, and other biomarkers are promising but are not available in daily care.
- For the evaluation of the disease activity of GCA, ESR and CRP are still relevant. During the first 3 months, the ESR might be more sensitive to diagnosing flares than the CRP. IL-6, IL-12, and IL-23 interferons are promising leads but are often unavailable in daily care.
- Some biomarkers have been suggested to predict a poor outcome for vascular complications, such as pentraxin-3 and anti-endothelin-1 receptor A autoantibodies. But once again, those markers are not available in daily care.
- A composite score of inflammatory parameters (fever, weight loss, ESR, hemoglobin, leukocytes, and thrombocytes) could be the most readily implemented predictor of the cumulative dose of GC in GCA, even though thresholds need to be validated.
- No biomarker is clearly identified to replace CRP in treatments that inhibit IL-6R. But osteopontin, C3, and C4 have promising published data.
- In PMR, most biomarkers have been studied in comparison to healthy controls and are mainly clues to pathophysiology.
- ESR, MMP-3, and the angiopoietin 1/angiopoietin 2 ratio demonstrated their abilities to identify underlying GCA in PMR patients.
- Several biomarkers (IL-6, TNFα, vascular endothelial growth factor, BAFF) correlate with CRP and/or ESR in PMR.
- Full blood count elements (high neutrophil-to-lymphocyte ratio, a high red blood cell distribution width, and a low hemoglobin) and sequential CRP measurements are still the main predictors of the response to GC in PMR.

**Table 5:** Summary of the studies evaluating the performances of biomarkers using a receiver operating characteristic curve analysis.

First author	Year	Disease	Disease group (n)	Stage of the disease	Control group	Control group (n)	Biomarkers studied	Sensitivity (%)	Specificity (%)	PPV (%)	NPV (%)	AUC	Threshold
CRP and ESR													
Kermani (23)	2012	GCA (TAB +)	587	Diagnosis	TAB negative	177	ESR	84.2	29.5	26.4	86.1	NA	NA
Oh (24)	2018	GCA (TAB positive)	84	Diagnosis	GCA look-alike (negative TAB)	289	ESR	NA	NA	NA	NA	0.67	>50 mm/h
Chan (25)	2019	GCA	139	Diagnosis	Suspected GCA	131	ESR	65.5	57.3	61.9	61.0	0.65	>50 mm/h
van Sleen (17)	2022	PMR with overlapping GCA (C1)	13	Diagnosis	Isolated PMR (C1)	25	ESR	85	64	NA	NA	0.82	>60 mm/h
van Sleen (17)	2022	PMR with overlapping GCA (cohort 2)	11	Diagnosis	Isolated PMR (cohort 2)	39	ESR	64	92	NA	NA	0.77	>91 mm/h
Atlas (26)	2023	GCA (TAB positive)	86	Diagnosis	TAB-negative	255	ESR	91.5	37.4	36.2	91.9	0.68	NA
Kermani (23)	2012	GCA (TAB +)	587	Diagnosis	TAB negative	177	CRP	86.4	30.5	27.2	88.6	NA	NA
Oh (24)	2018	GCA (TAB positive)	79	Diagnosis	GCA look-alike (negative TAB)	294	CRP	NA	NA	NA	NA	0.63	≥24.5 mg/l
Chan (25)	2019	GCA	139	Diagnosis	Suspected GCA	131	CRP	66.9	67.9	68.9	65.9	0.72	>20 mg/l
Hattori	2020	PMR in remission after 24 months or less GC therapy	20	After 1 month of treatment	PMR not in remission after 24 months or less GC therapy	30	CRP	75	69	NA	NA	0.711	1.7 mg/l
van Sleen (17)	2022	PMR with overlapping GCA (C1)	13	Diagnosis	Isolated PMR (C1)	25	CRP	92	52	NA	NA	0.63	>13 mg/l
van Sleen (17)	2022	PMR with overlapping GCA (cohort 2)	11	Diagnosis	Isolated PMR (cohort 2)	39	CRP	100	27	NA	NA	0.63	>13 mg/l
van Sleen (17)	2022	GCA (cohort 1)	52	Untreated	GCA look-alike	18	CRP	72	62	NA	NA	0.63	>62 mg/l
van Sleen (17)	2022	GCA (cohort 2)	48	Untreated	Infection	16	CRP	69	50	NA	NA	0.58	>52 mg/l
Atlas (26)	2023	GCA (TAB positive)	86	Diagnosis	TAB-negative	255	CRP	96.2	41.3	38.3	96.6	0.76	NA
Kermani (23)	2012	GCA (TAB +)	587	Diagnosis	TAB negative	177	ESR and CRP	80.8	41.2	29.3	87.7	NA	NA
Atlas (26)	2023	GCA (TAB positive)	86	Diagnosis	TAB-negative	255	ESR and CRP	91.0	51.2	40.0	93.6	NA	NA
Atlas (26)	2023	GCA (TAB positive)	86	Diagnosis	TAB-negative	255	ESR or CRP	96.2	27.4	33.9	94.8	NA	NA
Hematological markers													
Oh (24)	2018	GCA (TAB positive)	90	Diagnosis	GCA look-alike (negative TAB)	324	NLR	NA	NA	NA	NA	0.55	>9.35.10^9^/l
Oh (24)	2018	GCA (TAB positive)	89	Diagnosis	GCA look-alike (negative TAB)	310	PLR	NA	NA	NA	NA	0.62	>370.10^9^/l
Oh (24)	2018	GCA (TAB positive)	89	Diagnosis	GCA look-alike (negative TAB)	310	Platelets	NA	NA	NA	NA	0.66	>400.10^9^/l
Chan (25)	2019	GCA	139	Diagnosis	Suspected GCA	131	Platelets	71.2	62.6	66.9	67.2	0.72	>300.10^9^/l
van Sleen (17)	2022	GCA (cohort 1)	52	Untreated	GCA look-alike	18	Platelets	72	63	NA	NA	0.72	>391.10^9^/l
van Sleen (17)	2022	GCA (cohort 2)	48	Untreated	Infection	16	Platelets	80	73	NA	NA	0.75	>318.10^9^/l
Atlas (26)	2023	GCA (TAB positive)	86	Diagnosis	TAB-negative	255	Platelets	51.9	79.3	47.6	82.0	0.71	NA
Atlas (26)	2023	GCA (TAB positive)	86	Diagnosis	TAB-negative	255	Hemoglobin	61.7	52.2	31.8	79.1	0.44	NA
Autoantibodies													
Liozon (29)	2000	Relapsing GCA	21	Under treatment	Unrelapsing GCA	21	Anticardiolipin AAb	74.2	100	NA	NA	NA	NA
Baerlecken (31)	2012	GCA and/or PMR	47	Untreated	HIV, tuberculosis, B-NHL, GPA, AS, PsA, blood donors	41	Ab to the ferritin heavy chain protein	NA	NA	64.9	NA	0.87	61.5 U/ml
Baerlecken (31)	2012	GCA and/or PMR	47	Untreated	NA	NA	Ab to the N-terminal 27 amino acids of the human ferritin heavy chain	NA	NA	91.7	NA	0.87	19.5 U/ml
Baerlecken (31)	2012	GCA and/or PMR	47	Untreated	NA	NA	Ab to the N-terminal part of the ferritin of *S epidermidis*	NA	NA	88.9	NA	0.83	4.9 U/ml
Régent (32)	2013	GCA (TAB+ and TAB-)	69	Diagnosis	GCA-look alike	47	IgG Ab directed against a peptide of the human ferritin heavy chain	NA	NA	71.9	56.9	NA	NA
Other markers													
van der Geest (21)	2015	GCA	12	Diagnosis	Healthy controls	13	BAFF	100	77	NA	NA	0.90	1,018 pg/ml
van der Geest (21)	2015	PMR	12	Diagnosis	Healthy controls	13	BAFF	92	69	NA	NA	0.83	989 pg/ml
van der Geest (21)	2015	GCA	12	Diagnosis	Healthy controls	13	CXCL9	91	100	NA	NA	0.93	55 pg/ml
van der Geest (21)	2015	PMR	12	Diagnosis	Healthy controls	13	CXCL9	100	77	NA	NA	0.97	37 pg/ml
van der Geest (21)	2015	PMR	12	Diagnosis	Healthy controls	13	CXCL10	NA	NA	NA	NA	0.85	NA
van der Geest (21)	2015	GCA	12	Diagnosis	Healthy controls	13	IL-6	92	100	NA	NA	0.98	7 pg/ml
van der Geest (21)	2015	PMR	12	Diagnosis	Healthy controls	13	IL-6	100	100	NA	NA	1.0	8 pg/ml
van Sleen (17)	2022	PMR with overlapping GCA (C1)	13	Diagnosis	Isolated PMR (C1)	25	Angpt2/Angpt1 ratio	85	64	NA	NA	0.78	>0.048
van Sleen (17)	2022	PMR with overlapping GCA (cohort 2)	11	Diagnosis	Isolated PMR (cohort 2)	39	Angpt2/Angpt1 ratio	100	72	NA	NA	0.88	>0.051
van Sleen (17)	2022	PMR with overlapping GCA (C1)	13	Diagnosis	Isolated PMR (C1)	25	MMP-3	69	92	NA	NA	0.81	<23 ng/ml
van Sleen (17)	2022	PMR with overlapping GCA (cohort 2)	11	Diagnosis	Isolated PMR (cohort 2)	39	MMP-3	80	83	NA	NA	0.82	<14 ng/ml
van Sleen (17)	2022	GCA (cohort 1)	52	Untreated	GCA look-alike	18	MMP-3	67	67	NA	NA	0.66	>18.6 ng/ml
van Sleen (17)	2022	GCA (cohort 2)	48	Untreated	Infection	16	MMP-3	92	70	NA	NA	0.83	>13.3 ng/ml
Fukui (102)	2016	PMR	115	Diagnosis	PMR with GCA	17	MMP-3	91	66	NA	NA	0.81	>140 ng/ml
Fukui (102)	2016	PMR	115	Diagnosis	GCA (with or without PMR)	29	MMP-3	91	73	NA	NA	0.86	>118.2 ng/ml
van Sleen (17)	2022	PMR with overlapping GCA (C1)	13	Diagnosis	Isolated PMR (C1)	25	sCD206	77	64	NA	NA	0.66	>191 ng/ml
van Sleen (17)	2022	PMR with overlapping GCA (cohort 2)	11	Diagnosis	Isolated PMR (cohort 2)	39	sCD206	80	62	NA	NA	0.71	>178 ng/ml
van Sleen (17)	2022	GCA (cohort 1)	52	Untreated	GCA look-alike	18	PR3	63	60	NA	NA	0.57	>38 ng/ml
van Sleen (17)	2022	GCA (cohort 2)	48	Untreated	Infection	16	PR3	92	61	NA	NA	0.79	>66 ng/ml
Prieto-González (54)	2017	GCA	76	Diagnosis	Healthy controls	25	Osteopontin	80	84	NA	NA	0.862	59.79 nd/dl
Prieto-González (98)	2017	GCA active patients		Follow-up	GCA inactive patients	NA	Osteopontin	77	78	NA	NA	0.836	67.28 ng/dl

AAb, autoantibodies; Ab, antibodies; Angpt, angiopoietin; AS, ankylosing spondylitis; BAFF, B-cell activating factor; B-NHL, B-Non Hodgkin’s lymphoma; CD, cluster of differentiation; CRP, C-reactive protein; CXCL, chemokine (C-X-C motif) ligand; ESR, erythrocyte sedimentation rate; GC, glucocorticoids; GCA, giant cell arteritis; GPA, granulomatosis with polyangeitis; HIV, human immunodeficience virus; MMP, matrix matalloproteinase; NLR, Neutrophil to lymphocyte ratio; NPV, negative predictive value; PLR, Platelet to lymphocyte ratio; PMR, polymyalgia rheumatica; PPV, positive predictive value; PsA, psoriatic arthritis; TAB, temporal artery biopsy.

NA, Not available.

**Table 6:** Summary of the studies evaluating the correlation between biomarkers and other clinical or biological outcomes.

First author	Year	Disease	Number of patients in the disease group	Stage of the disease	Biomarker	Correlated parameter	Correlation coefficient	P value
Biomarkers from daily biology								
Fukui (102)	2016	GCA	29	Diagnosis	CRP	ESR	0.41	Significant
Fukui (102)	2016	PMR	115	Diagnosis	CRP	ESR	0.62	Significant
van Sleen (37)	2019	GCA	42	Diagnosis	CRP	ESR	0.80	<0.01
van Sleen (37)	2019	PMR	31	Diagnosis	CRP	ESR	0.36	NS
Jud (78)	2023	GCA	20	Treated patients	CRP	Diameter of the thoracic descending aorta	0.519	0.019
Jud (78)	2023	GCA	20	Treated patients	Lymphocyte count	Diameter of the thoracic descending aorta	0.504	0.023
van Sleen (37)	2019	GCA	42	Diagnosis	Hemoglobin	ESR	-0.51	<0.01
van Sleen (37)	2019	PMR	31	Diagnosis	Hemoglobin	ESR	-0.65	<0.01
van Sleen (37)	2019	GCA	42	Diagnosis	Platelets	CRP	0.49	<0.01
van Sleen (37)	2019	GCA	42	Diagnosis	Platelets	ESR	0.54	<0.01
Jung (106)	2019	PMR	94	Diagnosis	NLR	ESR	-0.03	0.767
Jung (106)	2019	PMR	94	Diagnosis	NLR	CRP	0.572	<0.001
Jung (106)	2019	PMR	94	Diagnosis	PLR	ESR	0.210	0.038
Jung (106)	2019	PMR	94	Diagnosis	PLR	CRP	0.424	<0.001
Jung (106)	2019	PMR	94	Diagnosis	MLR	ESR	0.177	0.082
Jung (106)	2019	PMR	94	Diagnosis	MLR	CRP	0.421	<0.001
Conticini (91)	2023	GCA	139	Diagnosis	C3	CRP	0.3	<0.01
Conticini (91)	2023	GCA	139	Diagnosis	C4	CRP	0.08	0.25
Conticini (91)	2023	GCA	139	Remission	C3	CRP	0.7	<0.01
Conticini (91)	2023	GCA	139	Remission	C4	CRP	0.66	<0.01
Conticini (91)	2023	GCA	139	Relapse	C3	CRP	0.68	0.01
Conticini (91)	2023	GCA	139	Relapse	C4	CRP	0.007	0.98
Conticini (91)	2023	GCA	139	Remission under tocilizumab	C3	CRP	-0.04	<0.9
Conticini (91)	2023	GCA	139	Remission under tocilizumab	C4	CRP	0.4	0.28
Cytokines								
Dasgupta (38)	1990	GCA and PMR	15	Untreated disease	IL-6	ESR	0.76	<0.05
Roche (39)	1993	GCA and PMR	31	Untreated disease	IL-6	ESR	0.20	NS
Roche (39)	1993	GCA and PMR	31	Untreated disease	IL-6	Platelets	0.10	NS
Hernández-Rodríguez (49)	2002	GCA	62	Diagnosis	IL-6	CRP	0.378	0.025
García-Martínez (116)	2010	GCA	54	Diagnosis	IL-6	CRP	0.296	0.03
van der Geest (21)	2015	GCA	12	Diagnosis	IL-6	CRP	0.73	<0.05
Pulsatelli (41)	2017	GCA	14	Untreated disease	IL-6	CRP	NA	NS
García-Martínez (116)	2010	GCA	54	Diagnosis	IL-6	ESR	0.078	NS
van der Geest (21)	2015	GCA	12	Diagnosis	IL-6	ESR	0.68	<0.05
Pulsatelli (41)	2017	GCA	14	Untreated disease	IL-6	ESR	NA	NS
van der Geest (21)	2015	PMR	12	Diagnosis	IL-6	ESR	0.76	<0.01
van der Geest (21)	2015	PMR	12	Diagnosis	IL-6	CRP	0.79	<0.01
Carvajal Alegria (108)	2021	PMR	18	Diagnosis	IL-6	Leukocytes	0.715	<0.001
Carvajal Alegria (108)	2021	PMR	18	Diagnosis	IL-6	Neutrophils	0.767	<0.001
Carvajal Alegria (108)	2021	PMR	18	Diagnosis	IL-6	Hemoglobin	0.575	0.012
Carvajal Alegria (108)	2021	PMR	18	Diagnosis	IL-6	Platelets	0.47	0.049
Pulsatelli (105)	2008	PMR	93	Diagnosis	sIL-6R	Number of relapses	0.334	0.01
Pulsatelli (105)	2008	PMR	93	After 1 month of treatment	sIL-6R	Number of relapses	0.246	0.023
Pulsatelli (105)	2008	PMR	93	After 3 months of treatment	sIL-6R	Number of relapses	0.253	0.021
Pulsatelli (105)	2008	PMR	93	After 12 months of treatment	sIL-6R	Number of relapses	0.233	0.035
Hernández-Rodríguez (49)	2002	GCA	62	Diagnosis	TNFα	ESR	0.364	0.018
García-Martínez (116)	2010	GCA	54	Diagnosis	TNF α	ESR	0.248	NS
García-Martínez (116)	2010	GCA	54	Diagnosis	TNF α	CRP	0.19	NS
Hernández-Rodríguez (49)	2002	GCA	62	Diagnosis	TNFα	Haptoglobin	0.448	0.022
Hernández-Rodríguez (49)	2002	GCA	62	Diagnosis	TNFα	Hemoglobin	-0.329	0.033
Espígol-Frigolé (51)	2013	GCA	57	Diagnosis	IL-17A mRNA *	ESR	0.0886	0.60
Espígol-Frigolé (51)	2013	GCA	57	Diagnosis	IL-17A mRNA *	Hemoglobin	-0.03563	0.82
Espígol-Frigolé (51)	2013	GCA	57	Diagnosis	IL-17A mRNA *	CRP	0.1495	0.45
Espígol-Frigolé (51)	2013	GCA	57	Diagnosis	IL-17A mRNA *	IL-6	0.363	0.025
Espígol-Frigolé (51)	2013	GCA	57	Diagnosis	IL-17A mRNA *	IL23p19 mRNA	0.397	0.008
Espígol-Frigolé (51)	2013	GCA	57	Diagnosis	IL-17A mRNA *	TGFβ	-0.037	0.848
Angiogenesis								
Meliconi (109)	2000	PMR	29	Diagnosis	VEGF	ESR	0.40	0.041
Meliconi (109)	2000	PMR	29	Diagnosis	VEGF	CRP	0.39	0.033
Baldini (19)	2012	GCA with ischemic complication	27	Follow-up	VEGF	ESR	0.672	0.001
Baldini (19)	2012	GCA with ischemic complication	27	Follow-up	VEGF	CRP	0.520	0.007
Dimitrijevic (75)	2010	GCA	10	Diagnosis	Endothelin-1*	ESR	NA	NS
Dimitrijevic (75)	2010	GCA	10	Diagnosis	Endothelin-1*	CRP	0.75	<0.05
Dimitrijevic (75)	2010	GCA	10	Diagnosis	Endothelin-B receptor*	ESR	NA	NS
Dimitrijevic (75)	2010	GCA	10	Diagnosis	Endothelin-B receptor*	CRP	0.65	<0.05
Klapa (77)	2019	GCA	42	NA	Anti-endohtelin-A receptor aAb	ESR	NA	NS
Klapa (77)	2019	GCA	42	NA	Anti-endohtelin-A receptor aAb	CRP	NA	NS
Klapa (77)	2019	GCA	42	NA	Anti-endohtelin-A receptor aAb	Hemoglobin	NA	NS
Tissue remodelling								
Prieto-González (54)	2017	GCA	76	Diagnosis	Osteopontin	ESR	0.32	0.009
Prieto-González (54)	2017	GCA	76	Diagnosis	Osteopontin	CRP	0.42	<0.001
Prieto-González (54)	2017	GCA	76	Diagnosis	Osteopontin	IL-6	0.34	0.005
Fukui (102)	2016	GCA	29	Diagnosis	MMP-3	CRP	0.27	NS
Fukui (102)	2016	PMR	115	Diagnosis	MMP-3	CRP	0.14	NS
Fukui (102)	2016	GCA	29	Diagnosis	MMP-3	ESR	-0.03	NS
Fukui (102)	2016	PMR	115	Diagnosis	MMP-3	ESR	0.17	NS
Other biomarkers								
Ellingsen (104)	2000	GCA	33	Diagnosis	MCP-1	ESR	0.008	0.97
Ellingsen (104)	2000	PMR	27	Diagnosis	MCP-1	ESR	0.06	0.77
Baldini (19)	2012	GCA with ischemic complication	27	Follow-up	PTX3	ESR	0.453	0.018
Baldini (19)	2012	GCA with ischemic complication	27	Follow-up	PTX3	CRP	0.530	0.005
van der Geest (21)	2015	GCA	12	Diagnosis	BAFF	CRP	0.85	<0.01
van der Geest (21)	2015	GCA	12	Diagnosis	BAFF	ESR	0.83	<0.01
van der Geest (21)	2015	PMR	12	Diagnosis	BAFF	CRP	0.77	<0.01
van der Geest (21)	2015	PMR	12	Diagnosis	BAFF	ESR	0.79	<0.01

aAb, autoantibodies; BAFF, B cell activating factor; ESR, erythrocyte sedimentation rate; GCA, giant cell arteritis; IL, interleukin; MCP, Monocyte chemoattractant protein; MLR, monocyte–lymphocyte ratio; NLR, neutrophil–lymphocyte ratio; PLR: platelet–lymphocyte ratio; PMR, polymyalgia rheumatica; TAB, temporal artery biopsy; TGF, transforming growth factor; TNF, tumor necrosis factor; VEGF, vascular endothelial growth factor.

*Evaluation in temporal artery biopsies.NA, Not available.

To obtain reliable biomarkers, the methodology of the studies is critical. For example, several studies were performed to identify biomarkers useful for the diagnosis of GCA or PMR but used healthy controls as a comparator. However, in daily practice, physicians do not need a test to discriminate patients from healthy people, but to discriminate patients with GCA (or PMR) from patients with other diseases mimicking GCA (or PMR). But few studies are done in such cohorts.

Many biomarkers are described as higher or lower in one population compared to another. But the comparison of the mean level of a marker in a subgroup does not mean that it enables us to properly discriminate those groups. Depending on the scattering of the data, a biomarker can have various properties. Again, in a specific context, one could be looking for a biomarker with high specificity, high sensitivity, or sometimes both. Performances, evaluated with ROC curves, and the definition of a threshold to establish sensitivity and specificity, are both elements mandatory to implement the use of a biomarker in clinical practice. **Table 7** presents the personal point of view and points to consider by the authors about studies on biomarkers in GCA and PMR.

**Table 7:** Points to consider in biomarkers studies.

Topic	Lines of thinking	Points to consider in GCA and PMR
Scientific question: What is the aim of the study?		
	Diagnosis	Diagnosis is a challenge in some patients, mainly when it comes to alternate diagnosis. The diagnosis of underlying vasculitis or underlying cancer in PMR might also be relevant.
	Prognosis	Prognosis might include the risk of relapses or complications
	Follow-up of disease activity	APR are currently used in association to clinical symptoms to follow disease activity. But, in some cases, APR cannot be used, and alternatives could be useful.
	Drug choice	GC remain the basis of treatment in both diseases. But with the growing number of targeted therapies evaluated, biomarkers could be useful to make a choice.
	Drug monitoring	Tapering GC without a risk of flare is a daily question. Some therapies interact with APR and disturb the monitoring of disease activity.
Choice of the biomarker(s) and of the technique		
	An isolated biomarker	Considering our current knowledge of the pathophysiology of the diseases, one could select a specific actor of inflammation, tissue remodeling, aging, etc. But now technology enables the concomitant analysis of many parameters, from dozens to thousands. In daily practice, such analyses are often not available. So, research based on large panels should aim at identifying only one or few biomarkers. The use of “signatures” is also possible, but rarely available in daily practice.
	A panel of selected biomarkers	
	A “omic” approach	
Choice of the biological material		
	Serum	The choice of the biological material should be guided by its availability for research, but also for generalization in daily practice. In GCA, temporal artery biopsies are still performed for diagnosis, but infiltrates are not always present, and the use imaging techniques has decreased the number of biopsies. In PMR, tissue is not routinely analyzed for patient’s care.
	Plasma	
	Isolated cells	
	Biopsy tissue	
Choice of the study population: Does the population fit the objective of the study?		
	Affected population	The stage of the disease (early disease, relapse) and the treatment intake (GC, csDMARDs, targeted therapies) must be considered.
	Control population	The control population might fit with the aim of the study and the possible use of the biomarker in daily practice. The control population might not be the same if the aim is to differentiate GCA or PMR from mimics, to differentiate PMR with or without an underlying vasculitis or to differentiate GCA or PMR patients with and without a relapse.
	Size of the populations	The size of the population might fit the number of explored biomarkers to ensure a proper power of analysis. The need for a validation cohort might be considered.
Choice of the type of analysis		
	ROC curves and discrimination performances	A difference in the mean of a biomarker between groups is often not sufficient for a clinical use. ROC curves, with an area under the curves, a threshold definition, calculation of sensitivity, specificity, positive predictive value, negative predictive value, should be considered.
	Scores	Biomarkers can be associated to each other or associated to clinical or imaging data to build scores, or probability tables.
	Validation cohort and second cohort	Many studies are performed on one cohort of patients. To confirm the usefulness of the identified biomarkers in a validation cohort. A cohort is sometimes split in two for discovery and validation. But a validation with a different cohort might confer more strength to the results.

APR, acute phase reactants; csDMARDs, conventional synthetic disease modifying anti-rheumatic drugs; GC, glucocorticoids.

Biomarkers can be used in an isolated manner; however, they are now more commonly used in associations with several biomarkers or with clinical characteristics to increase their performance. In other diseases, several matrices have been proposed to aid the disease prognosis. In cardiology, such matrices, including, among others, age, gender, blood pressure, and lipids, have been used for many years (117). In inflammatory rheumatism, such matrices have also been developed, for example, in rheumatoid arthritis (118) or spondyloarthritis (119). In GCA and PMR, one study did propose the use of a set of associated biomarkers, but no matrix for prediction has been established (18). Associations of biomarkers might help stratify patients into subgroups at higher or lower risk of relapse and help adapt the treatment (120).

To conclude, ESR and CRP are still key elements in the diagnosis and monitoring of GCA and PMR. But two gray areas are still important. First, CRP and ESR do not enable a clear identification of the patients at risk of vascular complications in GCA. Second, if molecules that inhibit IL-6R are used, CRP and ESR are known to be artificially low and useless in most cases. Now that candidates have been identified in both situations, efforts should be made to convert and validate these biomarkers.

## Author contributions

All authors listed have made a substantial, direct, and intellectual contribution to the work, and approved it for publication.

## References

[1] Hemmig AK Gozzoli D Werlen L Ewald H Aschwanden M Blockmans D. Subclinical giant cell arteritis in new onset polymyalgia rheumatica a systematic review and meta-analysis of individual patient data. Semin Arthritis Rheumatism (2022) 55:152017. doi: 10.1016/j.semarthrit.2022.152017 35537222

[2] Weyand CM Ma-Krupa W Goronzy JJ. Immunopathways in giant cell arteritis and polymyalgia rheumatica. Autoimmun Rev (2004) 3:46–53. doi: 10.1016/S1568-9972(03)00064-8 14871649

[3] Guggino G Ferrante A Macaluso F Triolo G Ciccia F. Pathogenesis of polymyalgia rheumatica. Reumatismo (2018) 70:10–7. doi: 10.4081/reumatismo.2018.1048 29589398

[4] Martinez-Taboada VM Alvarez L RuizSoto M Marin-Vidalled MJ Lopez-Hoyos M. Giant cell arteritis and polymyalgia rheumatica: role of cytokines in the pathogenesis and implications for treatment. Cytokine (2008) 44:207–20. doi: 10.1016/j.cyto.2008.09.004 18986814

[5] Buttgereit F Dejaco C Matteson EL Dasgupta B. Polymyalgia rheumatica and giant cell arteritis: a systematic review. JAMA (2016) 315:2442. doi: 10.1001/jama.2016.5444 27299619

[6] Buttgereit F Matteson EL Dejaco C. Polymyalgia rheumatica and giant cell arteritis. JAMA (2020) 324:993. doi: 10.1001/jama.2020.10155 32897333

[7] Salvarani C Pipitone N Versari A Hunder GG. Clinical features of polymyalgia rheumatica and giant cell arteritis. Nat Rev Rheumatol (2012) 8:509–21. doi: 10.1038/nrrheum.2012.97 22825731

[8] Carvajal Alegria G van Sleen Y Graver JC Sandovici M Devauchelle-Pensec V Brouwer E. Aortic involvement in giant cell arteritis. Joint Bone Spine (2021) 88:105045. doi: 10.1016/j.jbspin.2020.06.018 32649986

[9] Camellino D Cimmino MA. Imaging of polymyalgia rheumatica: indications on its pathogenesis, diagnosis and prognosis. Rheumatology (2012) 51:77–86. doi: 10.1093/rheumatology/keq450 21565899

[10] Huwart A Garrigues F Jousse-Joulin S Marhadour T Guellec D Cornec D. Ultrasonography and magnetic resonance imaging changes in patients with polymyalgia rheumatica treated by tocilizumab. Arthritis Res Ther (2018) 20:11. doi: 10.1186/s13075-017-1499-2 29370856 PMC5785834

[11] Mackie SL Koduri G Hill CL Wakefield RJ Hutchings A Loy C. Accuracy of musculoskeletal imaging for the diagnosis of polymyalgia rheumatica: systematic review. RMD Open (2015) 1:e000100. doi: 10.1136/rmdopen-2015-000100 26535139 PMC4623371

[12] Van Der Geest KSM Treglia G Glaudemans AWJM Brouwer E Jamar F Slart RHJA. Diagnostic value of [18F]FDG-PET/CT in polymyalgia rheumatica: a systematic review and meta-analysis. Eur J Nucl Med Mol Imaging (2021) 48:1876–89. doi: 10.1007/s00259-020-05162-6 PMC8113217 33372248

[13] Writing group Reviewer group Members of EANM Cardiovascular Members of EANM Infection & Inflammation Members of Committees SNMMI Cardiovascular. FDG-PET/CT(A) imaging in large vessel vasculitis and polymyalgia rheumatica: joint procedural recommendation of the EANM, SNMMI, and the PET interest group (PIG), and endorsed by the ASNC. Eur J Nucl Med Mol Imaging (2018) 45:1250–69. doi: 10.1007/s00259-018-3973-8 PMC5954002 29637252

[14] Delaval L Samson M Schein F Agard C Tréfond L Deroux A. Temporal arteritis revealing antineutrophil cytoplasmic antibody-associated vasculitides: a case-control study. Arthritis Rheumatol (2021) 73:286–94. doi: 10.1002/art.41527 32951354

[15] Dejaco C Ramiro S Duftner C Besson FL Bley TA Blockmans D. EULAR recommendations for the use of imaging in large vessel vasculitis in clinical practice. Ann Rheum Dis (2018) 77:636–43. doi: 10.1136/annrheumdis-2017-212649 29358285

[16] Burja B Feichtinger J Lakota K Thallinger GG Sodin-Semrl S Kuret T. Utility of serological biomarkers for giant cell arteritis in a large cohort of treatment-naïve patients. Clin Rheumatol (2019) 38:317–29. doi: 10.1007/s10067-018-4240-x 30143961

[17] Van Sleen Y Therkildsen P Nielsen BD van der Geest KSM Hansen I Heeringa P. Angiopoietin-2/-1 ratios and MMP-3 levels as an early warning sign for the presence of giant cell arteritis in patients with polymyalgia rheumatica. Arthritis Res Ther (2022) 24:65. doi: 10.1186/s13075-022-02754-5 35255968 PMC8900446

[18] van Sleen Y Boots AMH Abdulahad WH Bijzet J Sandovici M van der Geest KSM. High angiopoietin-2 levels associate with arterial inflammation and long-term glucocorticoid requirement in polymyalgia rheumatica. Rheumatology (2020) 59(1):176–84. doi: 10.1093/rheumatology/kez261 31292652

[19] Baldini M Maugeri N Ramirez GA Giacomassi C Castiglioni A Prieto-González S. Selective up-regulation of the soluble pattern-recognition receptor pentraxin 3 and of vascular endothelial growth factor in giant cell arteritis: relevance for recent optic nerve ischemia: selective up-regulation of PTX3 and VEGF in GCA. Arthritis Rheumatism (2012) 64:854–65. doi: 10.1002/art.33411 21989653

[20] Kuret T Frank-Bertoncelj M Lakota K Žigon P Thallinger GG Kopitar AN. From active to non-active giant cell arteritis: longitudinal monitoring of patients on glucocorticoid therapy in combination with leflunomide. Front Med (Lausanne) (2021) 8:827095. doi: 10.3389/fmed.2021.827095 35127774 PMC8811148

[21] Van Der Geest KSM Abdulahad WH Rutgers A Horst G Bijzet J Arends S. Serum markers associated with disease activity in giant cell arteritis and polymyalgia rheumatica. Rheumatology (2015) 54:1397–402. doi: 10.1093/rheumatology/keu526 25724206

[22] Wadström K Jacobsson LTH Mohammad AJ Warrington KJ Matteson EL Jakobsson ME. Analyses of plasma inflammatory proteins reveal biomarkers predictive of subsequent development of giant cell arteritis: a prospective study. Rheumatol (Oxford) (2023) 62(6):2304–11. doi: 10.1093/rheumatology/keac581 PMC10234188 36255228

[23] Kermani TA Schmidt J Crowson CS Ytterberg SR Hunder GG Matteson EL. Utility of erythrocyte sedimentation rate and c-reactive protein for the diagnosis of giant cell arteritis. Semin Arthritis Rheumatism (2012) 41:866–71. doi: 10.1016/j.semarthrit.2011.10.005 PMC3307891 22119103

[24] Oh LJ Wong E Andrici J McCluskey P Smith JEH Gill AJ. Full blood count as an ancillary test to support the diagnosis of giant cell arteritis: full blood count in giant cell arteritis. Intern Med J (2018) 48:408–13. doi: 10.1111/imj.13713 29236347

[25] Chan FLY Lester S Whittle SL Hill CL. The utility of ESR, CRP and platelets in the diagnosis of GCA. BMC Rheumatol (2019) 3:14. doi: 10.1186/s41927-019-0061-z 31008443 PMC6456976

[26] Atlas IS Colley SM Chia MA. Utility of biomarkers and temporal artery biopsy length for investigating giant cell arteritis in Western Australia. Int J Rheum Dis (2023) 26:286–91. doi: 10.1111/1756-185X.14488 PMC10098702 36401819

[27] O’Neill L Rooney P Molloy D Connolly M McCormick J McCarthy G. Regulation of inflammation and angiogenesis in giant cell arteritis by acute-phase serum amyloid a. Arthritis Rheumatol (2015) 67:2447–56. doi: 10.1002/art.39217 26016600

[28] Duhaut P Berruyer M Pinede L Demolombe-Rague S Loire R Seydoux D. Groupe de recherche sur l’Artérite á cellules géantes. anticardiolipin antibodies and giant cell arteritis: a prospective, multicenter case-control study. Arthritis Rheumatism (1998) 41:701–9. doi: 10.1002/1529-0131(199804)41:4<701::AID-ART18>3.0.CO;2-P 9550480

[29] Liozon E Roblot P Paire D Loustaud V Liozon F Vidal E. Anticardiolipin antibody levels predict flares and relapses in patients with giant-cell (temporal) arteritis. a longitudinal study of 58 biopsy-proven cases. Rheumatol (Oxford) (2000) 39:1089–94. doi: 10.1093/rheumatology/39.10.1089 11035128

[30] Espinosa G Tàssies D Font J Muñoz-Rodríguez FJ Cervera R Ordinas A. Antiphospholipid antibodies and thrombophilic factors in giant cell arteritis. Semin Arthritis Rheumatism (2001) 31:12–20. doi: 10.1053/sarh.2001.23499 11503135

[31] Baerlecken NT Linnemann A Gross WL Moosig F Vazquez-Rodriguez TR Gonzalez-Gay MA. Association of ferritin autoantibodies with giant cell arteritis/polymyalgia rheumatica. Ann Rheum Dis (2012) 71:943–7. doi: 10.1136/annrheumdis-2011-200413 22228484

[32] Régent A Ly KH Blet A Agard C Puéchal X Tamas N. Contribution of antiferritin antibodies to diagnosis of giant cell arteritis. Ann Rheum Dis (2013) 72:1269–70. doi: 10.1136/annrheumdis-2012-202963 23576711

[33] Kuret T Lakota K Hočevar A Burja B Čučnik S Sodin-Semrl S. Evaluating the utility of autoantibodies for disease activity and relapse in giant cell arteritis. J Biol Regul Homeost Agents (2018) 32:313–9. 29685012

[34] Jiemy WF van Sleen Y van der Geest KS Ten Berge HA Abdulahad WH Sandovici M. Distinct macrophage phenotypes skewed by local granulocyte macrophage colony-stimulating factor (GM-CSF) and macrophage colony-stimulating factor (M-CSF) are associated with tissue destruction and intimal hyperplasia in giant cell arteritis. Clin Transl Immunol (2020) 9:e1164. doi: 10.1002/cti2.1164 PMC7453134 32884747

[35] van Sleen Y Jiemy WF Pringle S van der Geest KSM Abdulahad WH Sandovici M. A distinct macrophage subset mediating tissue destruction and neovascularization in giant cell arteritis: implication of the YKL-40/Interleukin-13 receptor α2 axis. Arthritis Rheumatol (2021) 73:2327–37. doi: 10.1002/art.41887 PMC9298326 34105308

[36] Grossman C Yassin N Avivi C Bornstein G Ben-Zvi I Barshack I. Cytokine expression in temporal arteries: comparative analysis between patients with biopsy-positive giant cell arteritis, biopsy-negative giant cell arteritis and biopsy-negative without arteritis. Clin Exp Rheumatol (2019) 37 Suppl 117:122–9. 31162032

[37] van Sleen Y Graver JC Abdulahad WH van der Geest KSM Boots AMH Sandovici M. Leukocyte dynamics reveal a persistent myeloid dominance in giant cell arteritis and polymyalgia rheumatica. Front Immunol (2019) 10:1981. doi: 10.3389/fimmu.2019.01981 31507597 PMC6714037

[38] Dasgupta B Panayi GS. Interleukin-6 in serum of patients with polymyalgia rheumatica and giant cell arteritis. Rheumatology (1990) 29:456–8. doi: 10.1093/rheumatology/29.6.456 2124160

[39] Roche NE Fulbright JW Wagner AD Hunder GG Goronzy JJ Weyand CM. Correlation of interleukin-6 production and disease activity in polymyalgia rheumatica and giant cell arteritis. Arthritis Rheumatism (1993) 36:1286–94. doi: 10.1002/art.1780360913 8216422

[40] Miler E Stapleton PP Mapplebeck S Mackerness C Gayford D Aung T. Circulating interleukin-6 as a biomarker in a randomized controlled trial of modified-release prednisone vs immediate-release prednisolone, in newly diagnosed patients with giant cell arteritis. Int J Rheum Dis (2019) 22:1900–4. doi: 10.1111/1756-185X.13702 31531960

[41] Pulsatelli L Boiardi L Assirelli E Pazzola G Muratore F Addimanda O. Interleukin-6 and soluble interleukin-6 receptor are elevated in large-vessel vasculitis: a cross-sectional and longitudinal study. Clin Exp Rheumatol (2017) 103(1):102–10. 28466804

[42] Conway R O’Neill L McCarthy GM Murphy CC Fabre A Kennedy S. Interleukin 12 and interleukin 23 play key pathogenic roles in inflammatory and proliferative pathways in giant cell arteritis. Ann Rheum Dis (2018) 77:1815–24. doi: 10.1136/annrheumdis-2018-213488 30097452

[43] Vieira M Régnier P Maciejewski-Duval A Le Joncour A Darasse-Jèze G Rosenzwajg M. Interferon signature in giant cell arteritis aortitis. J Autoimmun (2022) 127:102796. doi: 10.1016/j.jaut.2022.102796 35123212

[44] Zhang H Watanabe R Berry GJ Tian L Goronzy JJ Weyand CM. Inhibition of JAK-STAT signaling suppresses pathogenic immune responses in medium and Large vessel vasculitis. Circulation (2018) 137:1934–48. doi: 10.1161/CIRCULATIONAHA.117.030423 PMC5930040 29254929

[45] Koster MJ Crowson CS Giblon RE Jaquith JM Duarte-García A Matteson EL. Baricitinib for relapsing giant cell arteritis: a prospective open-label 52-week pilot study. Ann Rheum Dis (2022) 81:861–7. doi: 10.1136/annrheumdis-2021-221961 PMC9592156 35190385

[46] Nuenninghoff DM Hunder GG Christianson TJH McClelland RL Matteson EL. Incidence and predictors of large-artery complication (aortic aneurysm, aortic dissection, and/or large-artery stenosis) in patients with giant cell arteritis: a population-based study over 50 years. Arthritis Rheumatism (2003) 48:3522–31. doi: 10.1002/art.11353 14674004

[47] Salvarani C Cimino L Macchioni P Consonni D Cantini F Bajocchi G. Risk factors for visual loss in an Italian population-based cohort of patients with giant cell arteritis. Arthritis Rheum (2005) 53:293–7. doi: 10.1002/art.21075 15818722

[48] Hernández-Rodríguez J Segarra M Vilardell C Sánchez M García-Martínez A Esteban M-J. Elevated production of interleukin-6 is associated with a lower incidence of disease-related ischemic events in patients with giant-cell arteritis: angiogenic activity of interleukin-6 as a potential protective mechanism. Circulation (2003) 107:2428–34. doi: 10.1161/01.CIR.0000066907.83923.32 12742994

[49] Hernández-Rodríguez J García-Martínez A Casademont J Filella X Esteban M-J López-Soto A. A strong initial systemic inflammatory response is associated with higher corticosteroid requirements and longer duration of therapy in patients with giant-cell arteritis: inflammatory response and corticosteroids in GCA. Arthritis Rheumatism (2002) 47:29–35. doi: 10.1002/art1.10161 11932875

[50] Nesher G Nesher R Mates M Sonnenblick M Breuer GS. Giant cell arteritis: intensity of the initial systemic inflammatory response and the course of the disease. Clin Exp Rheumatol (2008) 26(3 Suppl 49):S30–4. 18799050

[51] Espígol-Frigolé G Corbera-Bellalta M Planas-Rigol E Lozano E Segarra M García-Martínez A. Increased IL-17A expression in temporal artery lesions is a predictor of sustained response to glucocorticoid treatment in patients with giant-cell arteritis. Ann Rheum Dis (2013) 72:1481–7. doi: 10.1136/annrheumdis-2012-201836 22993227

[52] Venhoff N Schmidt WA Bergner R Rech J Unger L Tony HP. op0182 secukinumab in giant cell arteritis: the randomised, parallel-group, double-blind, placebo-controlled, multicentre phase 2 titain trial. Ann Rheumatic Dis (2022) 81:121–2. doi: 10.1136/annrheumdis-2022-eular.806

[53] Estupiñán-Moreno E Ortiz-Fernández L Li T Hernández-Rodríguez J Ciudad L Andrés-León E. Methylome and transcriptome profiling of giant cell arteritis monocytes reveals novel pathways involved in disease pathogenesis and molecular response to glucocorticoids. Ann Rheum Dis (2022) 81:1290–300. doi: 10.1136/annrheumdis-2022-222156 PMC9380516 35705375

[54] Prieto-González S Terrades-García N Corbera-Bellalta M Planas-Rigol E Miyabe C Alba MA. Serum osteopontin: a biomarker of disease activity and predictor of relapsing course in patients with giant cell arteritis. potential clinical usefulness in tocilizumab-treated patients. RMD Open (2017) 3:e000570. doi: 10.1136/rmdopen-2017-000570 29299342 PMC5743901

[55] Hattori K Hirano Y Kojima T. Predictors of glucocorticoid-free remission in patients with polymyalgia rheumatica treated with prednisolone. Int J Rheum Dis (2020) 23:1581–6. doi: 10.1111/1756-185X.13978 32996694

[56] Restuccia G Boiardi L Cavazza A Catanoso M Macchioni P Muratore F. Flares in biopsy-proven giant cell arteritis in northern Italy: characteristics and predictors in a long-term follow-up study. Med (Baltimore) (2016) 95:e3524. doi: 10.1097/MD.0000000000003524 PMC4902491 27175649

[57] Samson M Devilliers H Ly KH Maurier F Bienvenu B Terrier B. Tocilizumab as an add-on therapy to glucocorticoids during the first 3 months of treatment of giant cell arteritis: a prospective study. Eur J Intern Med (2018) 57:96–104. doi: 10.1016/j.ejim.2018.06.008 30054122

[58] Alba MA García-Martínez A Prieto-González S Tavera-Bahillo I Corbera-Bellalta M Planas-Rigol E. Relapses in patients with giant cell arteritis: prevalence, characteristics, and associated clinical findings in a longitudinally followed cohort of 106 patients. Med (Baltimore) (2014) 93:194–201. doi: 10.1097/MD.0000000000000033 PMC4602452 25181312

[59] Bellan M Puta E Croce A Sacchetti GM Orsini F Zecca E. Role of positron emission tomography in the assessment of disease burden and risk of relapse in patients affected by giant cell arteritis. Clin Rheumatol (2020) 39:1277–81. doi: 10.1007/s10067-019-04808-7 31713732

[60] Muratore F Boiardi L Restuccia G Cavazza A Catanoso M Macchioni P. Relapses and long-term remission in large vessel giant cell arteritis in northern Italy: characteristics and predictors in a long-term follow-up study. Semin Arthritis Rheum (2020) 50:549–58. doi: 10.1016/j.semarthrit.2020.04.004 32446023

[61] Sugihara T Hasegawa H Uchida HA Yoshifuji H Watanabe Y Amiya E. Associated factors of poor treatment outcomes in patients with giant cell arteritis: clinical implication of large vessel lesions. Arthritis Res Ther (2020) 22:72. doi: 10.1186/s13075-020-02171-6 32264967 PMC7137303

[62] Labarca C Koster MJ Crowson CS Makol A Ytterberg SR Matteson EL. Predictors of relapse and treatment outcomes in biopsy-proven giant cell arteritis: a retrospective cohort study. Rheumatol (Oxford) (2016) 55:347–56. doi: 10.1093/rheumatology/kev348 PMC4939727 26385368

[63] Martinez-Lado L Calviño-Díaz C Piñeiro A Dierssen T Vazquez-Rodriguez TR Miranda-Filloy JA. Relapses and recurrences in giant cell arteritis: a population-based study of patients with biopsy-proven disease from northwestern Spain. Med (Baltimore) (2011) 90:186–93. doi: 10.1097/MD.0b013e31821c4fad 21512412

[64] Hocevar A Rotar Z Jese R Semrl SS Pizem J Hawlina M. Do early diagnosis and glucocorticoid treatment decrease the risk of permanent visual loss and early relapses in giant cell arteritis: a prospective longitudinal study. Med (Baltimore) (2016) 95:e3210. doi: 10.1097/MD.0000000000003210 PMC4998766 27057850

[65] Hachulla E Boivin V Pasturel-Michon U Fauchais AL Bouroz-Joly J Perez-Cousin M. Prognostic factors and long-term evolution in a cohort of 133 patients with giant cell arteritis. Clin Exp Rheumatol (2001) 19:171–6. 11326479

[66] Armstrong AT Tyler WB Wood GC Harrington TM. Clinical importance of the presence of giant cells in temporal arteritis. J Clin Pathol (2008) 61:669–71. doi: 10.1136/jcp.2007.049049 18326013

[67] Breuer GS Nesher R Reinus K Nesher G. Association between histological features in temporal artery biopsies and clinical features of patients with giant cell arteritis. Isr Med Assoc J (2013) 15:271–4. 23882888

[68] Hernández-Rodríguez J Segarra M Vilardell C Sánchez M García-Martínez A Esteban MJ. Tissue production of pro-inflammatory cytokines (IL-1beta, TNFalpha and IL-6) correlates with the intensity of the systemic inflammatory response and with corticosteroid requirements in giant-cell arteritis. Rheumatol (Oxford) (2004) 43:294–301. doi: 10.1093/rheumatology/keh058 14679293

[69] García-Martínez A Hernández-Rodríguez J Espígol-Frigolé G Prieto-González S Butjosa M Segarra M. Clinical relevance of persistently elevated circulating cytokines (tumor necrosis factor alpha and interleukin-6) in the long-term followup of patients with giant cell arteritis. Arthritis Care Res (Hoboken) (2010) 62:835–41. doi: 10.1002/acr.20043 20535794

[70] Baldini M Maugeri N Ramirez GA Giacomassi C Castiglioni A Prieto-González S. Selective up-regulation of the soluble pattern-recognition receptor pentraxin 3 and of vascular endothelial growth factor in giant cell arteritis: relevance for recent optic nerve ischemia. Arthritis Rheumatism (2012) 64:1487–7. doi: 10.1002/art.34515 21989653

[71] Pulsatelli L Peri G Macchioni P Boiardi L Salvarani C Cantini F. Serum levels of long pentraxin PTX3 in patients with polymyalgia rheumatica. Clin Exp Rheumatol (2010) 28:756–8. 20822713

[72] Pulsatelli L Boiardi L Assirelli E Pazzola G Muratore F Addimanda O. Imbalance between angiogenic and anti-angiogenic factors in sera from patients with large-vessel vasculitis. Clin Exp Rheumatol (2020) 124(2):23–30. 31573481

[73] Planas-Rigol E Terrades-Garcia N Corbera-Bellalta M Lozano E Alba MA Segarra M. Endothelin-1 promotes vascular smooth muscle cell migration across the artery wall: a mechanism contributing to vascular remodelling and intimal hyperplasia in giant-cell arteritis. Ann Rheum Dis (2017) 76:1624–34. doi: 10.1136/annrheumdis-2016-210792 28606962

[74] Lozano E Segarra M Corbera-Bellalta M García-Martínez A Espígol-Frigolé G Plà-Campo A. Increased expression of the endothelin system in arterial lesions from patients with giant-cell arteritis: association between elevated plasma endothelin levels and the development of ischaemic events. Ann Rheum Dis (2010) 69:434–42. doi: 10.1136/ard.2008.105692 19289383

[75] Dimitrijevic I Andersson C Rissler P Edvinsson L. Increased tissue endothelin-1 and endothelin-b receptor expression in temporal arteries from patients with giant cell arteritis. Ophthalmology (2010) 117:628–36. doi: 10.1016/j.ophtha.2009.07.043 20036012

[76] Régent A Ly KH Groh M Khifer C Lofek S Clary G. Molecular analysis of vascular smooth muscle cells from patients with giant cell arteritis: targeting endothelin-1 receptor to control proliferation. Autoimmun Rev (2017) 16:398–406. doi: 10.1016/j.autrev.2017.02.006 28232168

[77] Klapa S Müller A Koch A Heidecke H Kähler W Junker J. Decreased endothelin receptor a autoantibody levels are associated with early ischaemic events in patients with giant-cell arteritis. Ann Rheum Dis (2019) 78:1443–4. doi: 10.1136/annrheumdis-2019-215341 31186255

[78] Jud P Verheyen N Stradner MH Dejaco C Szolar D Thonhofer R. Association of immunological parameters with aortic dilatation in giant cell arteritis: a cross-sectional study. Rheumatol Int (2022) 43:477–85. doi: 10.1007/s00296-022-05186-1 PMC9968266 35996028

[79] Buttgereit F Matteson EL Dejaco C Dasgupta B. Prevention of glucocorticoid morbidity in giant cell arteritis. Rheumatol (Oxford) (2018) 57:ii11–21. doi: 10.1093/rheumatology/kex459 29982779

[80] Albrecht K Huscher D Buttgereit F Aringer M Hoese G Ochs W. Long-term glucocorticoid treatment in patients with polymyalgia rheumatica, giant cell arteritis, or both diseases: results from a national rheumatology database. Rheumatol Int (2018) 38:569–77. doi: 10.1007/s00296-017-3874-3 29124397

[81] Unizony S Arias-Urdaneta L Miloslavsky E Arvikar S Khosroshahi A Keroack B. Tocilizumab for the treatment of large-vessel vasculitis (giant cell arteritis, takayasu arteritis) and polymyalgia rheumatica. Arthritis Care Res (Hoboken) (2012) 64:1720–9. doi: 10.1002/acr.21750 22674883

[82] Işık M Kılıç L Doğan İ Calgüneri M. Tocilizumab for giant cell arteritis: an amazing result. Rheumatol Int (2013) 33:2961–2. doi: 10.1007/s00296-012-2516-z 22965672

[83] Lurati A Bertani L Re KA Marrazza M Bompane D Scarpellini M. Successful treatment of a patient with giant cell vasculitis (horton arteritis) with tocilizumab a humanized anti-interleukin-6 receptor antibody. Case Rep Rheumatol (2012) 2012:639612. doi: 10.1155/2012/639612 22937454 PMC3420619

[84] Kieffer P Hinschberger O Ciobanu E Jaeger-Bizet F Drabo A Mostoufizadeh T. Clinical and biological efficacy of tocilizumab in giant cell arteritis: report of three patients and literature review. Rev Med Interne (2014) 35:56–9. doi: 10.1016/j.revmed.2012.12.012 24075627

[85] Stone JH Tuckwell K Dimonaco S Klearman M Aringer M Blockmans D. Trial of tocilizumab in giant-cell arteritis. N Engl J Med (2017) 377:317–28. doi: 10.1056/NEJMoa1613849 28745999

[86] Stone JH Tuckwell K Dimonaco S Klearman M Aringer M Blockmans D. Glucocorticoid dosages and acute-phase reactant levels at giant cell arteritis flare in a randomized trial of tocilizumab. Arthritis Rheumatol (2019) 71:1329–38. doi: 10.1002/art.40876 PMC6772126 30835950

[87] Calderón-Goercke M Castañeda S Aldasoro V Villa I Prieto-Peña D Atienza-Mateo B. Tocilizumab in giant cell arteritis: differences between the GiACTA trial and a multicentre series of patients from the clinical practice. Clin Exp Rheumatol (2020) 124(2):112–19. 32441643

[88] Calderón-Goercke M Loricera J Aldasoro V Castañeda S Villa I Humbría A. Tocilizumab in giant cell arteritis. observational, open-label multicenter study of 134 patients in clinical practice. Semin Arthritis Rheumatism (2019) 49:126–35. doi: 10.1016/j.semarthrit.2019.01.003 30655091

[89] Miyabe C Miyabe Y Strle K Kim ND Stone JH Luster AD. An expanded population of pathogenic regulatory T cells in giant cell arteritis is abrogated by IL-6 blockade therapy. Ann Rheum Dis (2017) 76:898–905. doi: 10.1136/annrheumdis-2016-210070 27927642 PMC5744591

[90] Berger CT Rebholz-Chaves B Recher M Manigold T Daikeler T. Serial IL-6 measurements in patients with tocilizumab-treated large-vessel vasculitis detect infections and may predict early relapses. Ann Rheum Dis (2019) 78:1012–4. doi: 10.1136/annrheumdis-2018-214704 30670375

[91] Conticini E Hellmich B Frediani B Csernok E Löffler C. Utility of serum complement factors C3 and C4 as biomarkers during therapeutic management of giant cell arteritis. Scand J Rheumatol (2023) 52(3):276–82. doi: 10.1080/03009742.2022.2047311 35383517

[92] Vaith P Maas D von Stackelberg G Peter HH. A new serological reaction in patients with polymyalgia rheumatica and/or giant cell (temporal) arteritis: deposition of complement C4 and C3 components on rat kidney structures detected by indirect immunofluorescence. Rheumatol Int (1986) 6:255–61. doi: 10.1007/BF00541316 3544156

[93] Chen R Ma L Lv P Lin J Li C Yan Y. Serum complement 3 is a potential biomarker for assessing disease activity in takayasu arteritis. Arthritis Res Ther (2021) 23:63. doi: 10.1186/s13075-021-02433-x 33627173 PMC7903686

[94] Schmidt WA. Ultrasound in the diagnosis and management of giant cell arteritis. Rheumatol (Oxford) (2018) 57:ii22–31. doi: 10.1093/rheumatology/kex461 29982780

[95] Maleszewski JJ Younge BR Fritzlen JT Hunder GG Goronzy JJ Warrington KJ. Clinical and pathological evolution of giant cell arteritis: a prospective study of follow-up temporal artery biopsies in 40 treated patients. Mod Pathol (2017) 30:788–96. doi: 10.1038/modpathol.2017.10 PMC5650068 28256573

[96] van der Geest KSM Sandovici M van Sleen Y Sanders J-S Bos NA Abdulahad WH. Review: what is the current evidence for disease subsets in giant cell arteritis? Arthritis Rheumatol (2018) 70:1366–76. doi: 10.1002/art.40520 PMC6175064 29648680

[97] Devauchelle-Pensec V Carvajal-Alegria G Dernis E Richez C Truchetet M-E Wendling D. Effect of tocilizumab on disease activity in patients with active polymyalgia rheumatica receiving glucocorticoid therapy: a randomized clinical trial. JAMA (2022) 328:1053–62. doi: 10.1001/jama.2022.15459 PMC12285571 36125471

[98] Bonelli M Radner H Kerschbaumer A Mrak D Durechova M Stieger J. Tocilizumab in patients with new onset polymyalgia rheumatica (PMR-SPARE): a phase 2/3 randomised controlled trial. Ann Rheum Dis (2022) 81(6):838–44. doi: 10.1136/annrheumdis-2021-221126 35210264

[99] Koster MJ Matteson EL Warrington KJ. Large-Vessel giant cell arteritis: diagnosis, monitoring and management. Rheumatol (Oxford) (2018) 57:ii32–42. doi: 10.1093/rheumatology/kex424 29982778

[100] Blockmans D De Ceuninck L Vanderschueren S Knockaert D Mortelmans L Bobbaers H. Repetitive 18-fluorodeoxyglucose positron emission tomography in isolated polymyalgia rheumatica: a prospective study in 35 patients. Rheumatology (2006) 46:672–7. doi: 10.1093/rheumatology/kel376 17114803

[101] Lund-Petersen A Voss A Laustrup H. PET-CT findings in patients with polymyalgia rheumatica without symptoms of cranial ischaemia. Dan Med J (2017) 64(10):A5410. 28975885

[102] Fukui S Nunokawa T Kobayashi S Kamei S Yokogawa N Takizawa Y. MMP-3 can distinguish isolated PMR from PMR with GCA: a retrospective study regarding PMR and GCA in Japan. Modern Rheumatol (2016) 26:259–64. doi: 10.3109/14397595.2015.1071304 26156043

[103] Michailidou D Johansson L Kuley R Wang T Hermanson P Rantapää-Dahlqvist S. Immune complex-mediated neutrophil activation in patients with polymyalgia rheumatica. Rheumatol (Oxford) (2022), keac722. doi: 10.1093/rheumatology/keac722 PMC10393430 36562570

[104] Ellingsen T. Monocyte chemoattractant protein 1 (MCP-1) in temporal arteritis and polymyalgia rheumatica. Ann Rheumatic Dis (2000) 59:775–80. doi: 10.1136/ard.59.10.775 PMC1753013 11005777

[105] Pulsatelli L Boiardi L Pignotti E Dolzani P Silvestri T Macchioni P. Serum interleukin-6 receptor in polymyalgia rheumatica: a potential marker of relapse/recurrence risk. Arthritis Rheum (2008) 59:1147–54. doi: 10.1002/art.23924 18668607

[106] Jung J Lee E Suh C Kim H. Neutrophil-to-lymphocyte ratio and platelet-to-lymphocyte ratio are associated with disease activity in polymyalgia rheumatica. J Clin Lab Anal (2019) 33(9):e23000. doi: 10.1002/jcla.23000 31402523 PMC6868401

[107] Owen CE McMaster C Liew DFL Leung JL Scott AM Buchanan RRC. Neutrophil to lymphocyte ratio predicts glucocorticoid resistance in polymyalgia rheumatica. Int J Rheum Dis (2021) 24:56–62. doi: 10.1111/1756-185X.14000 33043616

[108] Carvajal Alegria G Cornec DYK Renaudineau Y Saraux A Devauchelle-Pensec V. Inflammatory markers are quickly improved by tocilizumab in early polymyalgia rheumatica and might predict early response to interleukin-6 blockade. Rheumatol Ther (2021) 8:751–60. doi: 10.1007/s40744-021-00299-8 PMC7981388 33745124

[109] Meliconi R Pulsatelli L Dolzani P Boiardi L Macchioni P Salvarani C. Vascular endothelial growth factor production in polymyalgia rheumatica. Arthritis Rheumatism (2000) 43:2472–80. doi: 10.1002/1529-0131(200011)43:11<2472::AID-ANR14>3.0.CO;2-B 11083270

[110] Cantini F Salvarani C Olivieri I Macchioni L Ranzi A Niccoli L. Erythrocyte sedimentation rate and c-reactive protein in the evaluation of disease activity and severity in polymyalgia rheumatica: a prospective follow-up study. Semin Arthritis Rheum (2000) 30:17–24. doi: 10.1053/sarh.2000.8366 10966209

[111] Myklebust G Gran JT. Prednisolone maintenance dose in relation to starting dose in the treatment of polymyalgia rheumatica and temporal arteritis. a prospective two-year study in 273 patients. Scand J Rheumatol (2001) 30:260–7. doi: 10.1080/030097401753180327 11727839

[112] Soddu D Sola D Bellan M Boin E Cittone MG Zecca E. Red cell distribution width is a potential predictor of early relapse in polymyalgia rheumatica. Reumatismo (2021) 73:117–21. doi: 10.4081/reumatismo.2021.1395 34342213

[113] Devauchelle-Pensec V Saraux L Berthelot JM De Bandt M Cornec D Guellec D. Assessing polymyalgia rheumatica activity when c-reactive protein is unavailable or uninterpretable. Rheumatol (Oxford) (2018) 57:666–70. doi: 10.1093/rheumatology/kex477 29346621

[114] Ribbens C. Increased matrix metalloproteinase-3 serum levels in rheumatic diseases: relationship with synovitis and steroid treatment. Ann Rheumatic Dis (2002) 61:161–6. doi: 10.1136/ard.61.2.161 PMC1753989 11796404

[115] Carvajal Alegria G Devauchelle-Pensec V Renaudineau Y Saraux A Pers J-O Cornec D. Correction of abnormal b-cell subset distribution by interleukin-6 receptor blockade in polymyalgia rheumatica. Rheumatol (Oxford) (2017) 56:1401–6. doi: 10.1093/rheumatology/kex169 28431111

[116] García-Martínez A Hernández-Rodríguez J Espígol-Frigolé G Prieto-González S Butjosa M Segarra M. Clinical relevance of persistently elevated circulating cytokines (tumor necrosis factor α and interleukin-6) in the long-term followup of patients with giant cell arteritis. Arthritis Care Res (2010) 62:835–41. doi: 10.1002/acr.20043 20535794

[117] Farzadfar F. Cardiovascular disease risk prediction models: challenges and perspectives. Lancet Global Health (2019) 7:e1288–9. doi: 10.1016/S2214-109X(19)30365-1 31488388

[118] Vanier A Smolen JS Allaart CF Van Vollenhoven R Verschueren P Vastesaeger N. An updated matrix to predict rapid radiographic progression of early rheumatoid arthritis patients: pooled analyses from several databases. Rheumatol (Oxford) (2020) 59:1842–52. doi: 10.1093/rheumatology/kez542 31722413

[119] Poddubnyy D Haibel H Listing J Märker-Hermann E Zeidler H Braun J. Baseline radiographic damage, elevated acute-phase reactant levels, and cigarette smoking status predict spinal radiographic progression in early axial spondylarthritis. Arthritis Rheum (2012) 64:1388–98. doi: 10.1002/art.33465 22127957

[120] Tomelleri A van der Geest KSM Sebastian A van Sleen Y Schmidt WA Dejaco C. Disease stratification in giant cell arteritis to reduce relapses and prevent long-term vascular damage. Lancet Rheumatol (2021) 3:e886–95. doi: 10.1016/S2665-9913(21)00277-0

